# Efficient persistent afterglow modulation using extended Indolo[2,3-*a*]carbazoles with six-membered rings in a polymer matrix

**DOI:** 10.1039/d5sc10031c

**Published:** 2026-03-06

**Authors:** Yu Lang, Jiahui Sun, Mengyao Jiang, Qingyun Jiang, Yongkang Du, Feng Wang, Jiadong Zhou, Guang Shi, Bingjia Xu, Cong Liu

**Affiliations:** a School of Chemistry, South China Normal University Guangzhou 510006 PR China zhoujd@scnu.edu.cn shg73@163.com liucong_011@163.com; b Kingfa Science & Technology Co., Ltd. Guangzhou 510663 China; c Zhuhai Vanteque Specialty Engineering Plastics Co., Ltd. Zhuhai 519050 China; d School of Environmental and Chemical Engineering, Wuyi University Jiangmen 529020 PR China

## Abstract

Organic persistent afterglow materials exhibit tremendous promise for applications in optoelectronic displays, anti-counterfeiting, and information encryption. Here, we introduce a structural modulation strategy in which a flexible cyclohexyl or rigid phenyl substituent is added to a 11,12-dihydroindolo[2,3-*a*]carbazole (ICz) scaffold, namely HICz and BICz, respectively, to precisely manipulate the (hybrid) afterglow emission, with a long lifetime and high efficiency in a melamine-formaldehyde (MF) polymer matrix. Flexible HICz promotes reverse intersystem crossing (RISC) for hybrid afterglow, whereas rigid BICz enlarges singlet-triplet energy gaps (Δ*E*_ST_), consequently driving the ISC process for pure phosphorescence afterglow, featuring an ultralong lifetime of 3.05 s and an impressive phosphorescence quantum yield of 17.59%. The afterglow duration for BICz-MF film exceeds 30 s in an ambient environment, substantially outperforming conventional ICz systems. Furthermore, a triplet-sensitized Förster resonance energy transfer (TS-FRET) strategy enables multicolor afterglow for optical encryption. This study establishes a generalizable molecular design principle for high-efficiency and long-afterglow organic materials.

## Abstract 2

Structure-modulated ICzs with flexible/rigid six-membered rings embedded in melamine-formaldehyde composites show efficient organic afterglow with a phosphorescence quantum yield (*Φ*_Phos._) of up to 17.59% and a lifetime (*τ*_Phos._) of up to 3.05 s.

## Graphical abstract

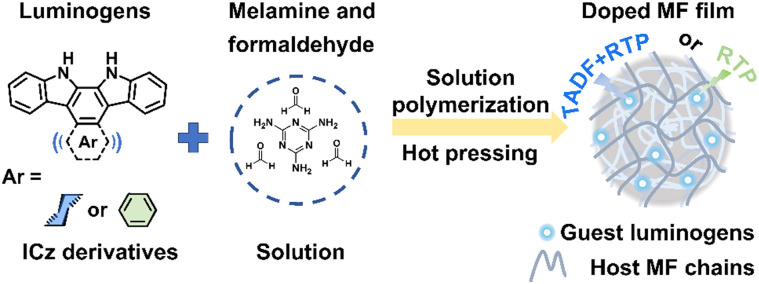

## Introduction

Ultralong afterglow materials have attracted enormous attention owing to their distinguished merit of long-lived emissive behavior, enabling advanced applications in bioimaging, information security, anti-counterfeiting, and organic light-emitting diodes (OLEDs).^1–10^ However, unlike inorganic phosphors or organometallic complexes, purely organic luminophores typically suffer from intrinsically weak spin–orbit coupling (SOC) and the rapid nonradiative deactivation of triplet excitons, leading to very weak phosphorescence under ambient conditions. To circumvent these limitations, various molecular design and material processing strategies, such as crystal engineering,^11–17^ host–guest doping,^18–23^ and supramolecular assembly,^24–26^ have been developed to enhance intersystem crossing (ISC) and stabilize triplet states, thereby enabling long-lived organic afterglow emission.^27–32^ Although substantial efforts have been devoted to rigidifying the molecular environment and suppressing nonradiative decay, achieving both ultralong phosphorescence lifetimes (>3 s) and high phosphorescence quantum yields (*Φ*_Phos_) (>15%) remains highly challenging (Tables S1 and S2).^33–40^

For room-temperature phosphorescence (RTP) materials, the ISC process generates meta-stable triplet excitons, which undergo radiative decay from the T_1_ to S_0_ state, resulting in afterglow emission with extended lifetimes. In contrast, for thermally activated delayed fluorescence (TADF) materials, a sufficiently minimized singlet-triplet energy gap (Δ*E*_ST_) enables efficient reverse intersystem crossing (RISC). The excitons harvest thermal energy to populate the S_1_ state, generating delayed fluorescence *via* S_1_ → S_0_ radiative decay. This dynamic control over ISC/RISC competition ultimately dictates the dominant afterglow pathway.^41,42^ Suppressed RISC directly favors radiative decay from the triplet state (*i.e.*, RTP), whereas accelerated RISC sustains efficient cycling between S_1_ and T_1_ to enable TADF. Currently, TADF design strategies primarily focus on minimizing Δ*E*_ST_, accelerating the RISC rate (*k*_RISC_), and balancing charge transfer.^43–45^ Consequently, although both TADF and RTP rely on manipulation of triplet excitons, they adopt opposing approaches to RISC regulation. TADF requires “activating” RISC to enable the upconversion of triplet excitons, while RTP necessitates “blocking” RISC to confine excitons within the triplet state for phosphorescent emission, and the suppression of nonradiative decay pathways from the triplet state is also essential for achieving RTP. Such precise tailoring of excited-state dynamics is the cornerstone of achieving diverse organic afterglow emission.

Among various organic persistent afterglow materials, 11,12-dihydroindolo[2,3-*a*]carbazole (ICz) has emerged as a particularly attractive molecular scaffold due to its rigid, planar heteroaromatic framework and outstanding chemical stability. These structural features are conducive to stabilizing triplet excitons and suppressing nonradiative deactivation, rendering ICz derivatives promising candidates for persistent luminescence (Table S3).^34,46–48^ To date, strategies for enhancing ICz-based afterglow performance have largely focused on conformational engineering, N-site functionalization, copolymerization, or isomer regulation. Tang *et al.* achieved an afterglow lifetime of 2.04 s and a quantum yield of 44.10% *via* conformational locking in PVA matrices.^34^ Xu *et al.* introduced a benzoic acid moiety at the N-position of ICz to obtain dual-mode emissive ICz-PVA films with a lifetime of 1.81 s and *Φ*_Phos_ of 19.80%.^47^ Ren *et al.* copolymerized ICz into a polyurethane-urea elastomer, producing a flexible afterglow material exhibiting a phosphorescence lifetime of 1.89 s.^48^ Despite these advances, ICz-based afterglow remains confined to short lifetimes (<3.0 s), primarily due to suboptimal ISC/RISC balance and incomplete suppression of triplet deactivation. Prior strategies relied exclusively on external rigidification or peripheral substitution and were thus incapable of directly modulating the intrinsic ISC and RISC processes of the chromophore.

**Fig. 1:**
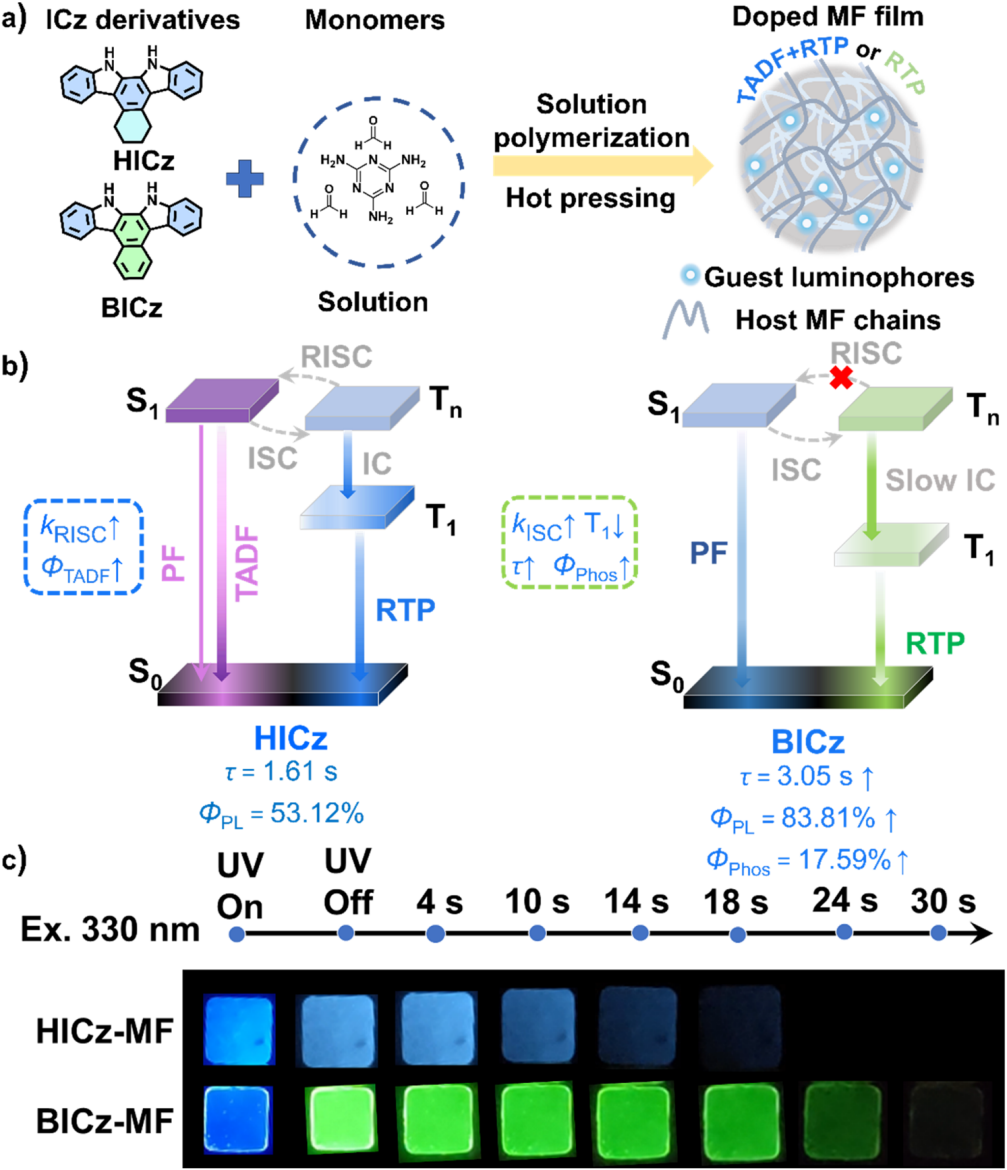
(a) The preparation process of MF film. (b) Simplified Jablonski diagrams describing the photophysical processes of fluorescence and phosphorescence. (c) Afterglow photographs of HICz-MF and BICz-MF in a dark room (*λ*_ex_ = 330 nm).

In this work, two ICz derivatives were designed and synthesized *via* the site-specific annulation of a six-membered ring at a previously unexplored position on the ICz core, and they were subsequently incorporated into a melamine-formaldehyde (MF) polymer matrix to achieve highly efficient persistent afterglow (Fig. 1a). 5,6,7,8,13,14-Hexahydrobenzo[*c*]-indolo[2,3-*a*]carbazole (HICz) incorporates a flexible cyclohexyl group and 13,14-dihydrobenzo[*c*]indolo[2,3-*a*]carbazole (BICz) contains a rigid phenyl π-extension. Although the cyclohexyl group of HICz promotes RISC-favorable conformations, its flexibility simultaneously accelerates nonradiative decay, resulting in a photoluminescence quantum yield (*Φ*_PL_) of only 53.12% for HICz, while that of BICz is 83.81%. Incorporating a rigid π-extended framework in BICz substantially facilitates the ISC process, thereby greatly amplifying the RTP contribution, with *Φ*_PL_ of 83.81%. The rigid π-extension in BICz not only facilitates the ISC process but also enlarges the singlet-triplet energy gap (Δ*E*_ST_). As a result, RISC is effectively suppressed, leading to purely RTP-dominated afterglow with an ultralong phosphorescence lifetime of 3.05 s and a high phosphorescence quantum yield (*Φ*_Phos_) of 17.59% (Fig. 1b). The superior phosphorescence performance and higher phosphorescence quantum yield of BICz-MF relative to HICz-MF translate directly into markedly longer afterglow durations. Upon UV excitation at 330 nm, the visible afterglow of HICz-MF persists for approximately 18 s, whereas BICz-MF exhibits an extended afterglow lasting over 30 s (Fig. 1c). Furthermore, by implementing a triplet-sensitized Förster resonance energy transfer (TS-FRET) strategy, multicolor afterglow emission was achieved, allowing for visually encrypted coding, anti-counterfeiting authentication, and hierarchical information storage, thereby significantly broadening the practical applicability of long-afterglow materials. Such exciting results indicate that this structural modulation strategy provides a molecular design principle for achieving efficient and long-lived organic afterglow materials.

## Results and discussion

The synthetic routes of HICz and BICz are depicted in Scheme S1. Briefly, HICz was synthesized *via* the coupling of two indole units with 2-chlorocyclohexanone, while BICz was prepared by coupling 2,3-diaminonaphthalene with two equivalents of 2-bromoiodobenzene, followed by debromination and intramolecular cyclization. The crude products were purified *via* silica-gel column chromatography and recrystallization to afford the target molecules in high purity. Their chemical structures were confirmed by ^1^H and ^13^C NMR spectroscopy and high-resolution mass spectrometry (HRMS) (Fig. S1–S9).^49^ Thermogravimetric analysis (TGA) revealed decomposition temperatures of 348 °C for HICz and 370 °C for BICz (Fig. S10), indicating excellent thermal stability, which is advantageous for subsequent material processing.

The photophysical properties of the solid powders of HICz and BICz were investigated. Both compounds exhibited deep-blue emission with maximum emission wavelengths (*λ*_em_) of 396 nm (HICz) and 378 nm (BICz) (Fig. S11a). Their prompt emission decayed rapidly, with short lifetimes of 6.23 and 24.46 ns (Fig. S11b), and long-lived emission was not detected. To evaluate their intrinsic excited-state properties, the molecules were dispersed in dilute tetrahydrofuran (THF) solution. The UV-vis absorption spectrum of HICz exhibited absorption bands at 271 and 325 nm, while BICz showed a slight redshift, with absorption peaks at 276 and 362 nm, indicating reduced HOMO–LUMO energy gaps with the extension of π-conjugation (Fig. S12a). The delayed emission spectra of HICz and BICz in dilute solutions at 77 K showed distinct peaks at 432 and 489 nm for HICz and BICz, respectively, which were attributed to phosphorescence. Moreover, the phosphorescence emission peaks showed almost unchanged positions at different concentrations (10^−3^–10^−6^ M) (Fig. S12c and d), confirming that the delayed emission was originated from monomeric phosphorescence rather than aggregated species.

**Fig. 2:**
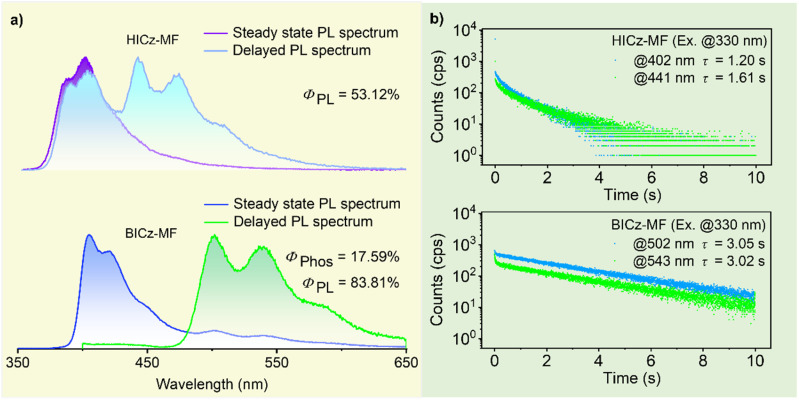
(a) Steady-state PL spectra and delayed PL spectra of HICz-MF and BICz-MF under ambient conditions. (b) Emission decay curves of HICz-MF and BICz-MF under ambient conditions (delay: 1 ms, *λ*_ex_ = 330 nm).

To decipher the intrinsic photophysical properties of the luminophores, the compounds were dispersed in a rigid MF polymer matrix to simultaneously minimize nonradiative relaxation and isolate environmental oxygen. As shown in Fig. S13, a series of MF-based composite films with dopant concentrations ranging from 0.01 to 0.50 wt% exhibited room-temperature afterglow emission. Upon increasing the dopant concentration, the afterglow lifetime initially increased and then decreased, with the optimum doping concentration being 0.05 wt%, which was used for subsequent studies (Fig. S14 and Table S4). As shown in Fig. 2a, in the steady-state PL spectra, both HICz-MF and BICz-MF films exhibited dominant emission peaks at about 400 nm, which were attributed to fluorescence bands consistent with those in dilute THF solution (Fig. S12b). Although HICz-MF and BICz-MF showed similar fluorescence features, their delayed emission profiles were quite different. HICz-MF exhibited dual delayed emission bands in the deep-blue region (*λ*_em_ = 383 nm and 402 nm) and blue region (*λ*_em_ = 441 nm and 470 nm), which might be attributed to delayed fluorescence and phosphorescence, respectively. In contrast, BICz-MF exhibited only a single phosphorescence band with fine structural peaks (*λ*_em_ = 502 nm and 543 nm), indicating purely RTP-dominated ultralong afterglow.

The afterglow lifetimes of HICz-MF and BICz-MF reached 1.61 and 3.05 s, respectively, whereas the blank MF film exhibited only negligible and short-lived afterglow (Fig. 2b and Fig. S15). Relative to the nanosecond-scale lifetimes observed for the powders, both molecules exhibited significantly prolonged lifetimes when doped into the MF matrix, which can be attributed to the suppression of nonradiative decay by the rigid, three-dimensional cross-linked polymer microenvironment. For HICz-MF, two delayed emission bands with lifetimes of 1.20 s (*λ*_em_ = 402 nm) and 1.61 s (*λ*_em_ = 441 nm) were observed. In contrast, BICz-MF exhibited two dominant afterglow emission peaks at 502 nm and 543 nm, with corresponding lifetimes of 3.05 and 3.02 s, respectively. These lifetimes surpassed those of most previously reported polymer-based afterglow systems (Tables S1 and S2).^33–40^ Excitation-dependent delayed emission measurements (250–360 nm) revealed consistent spectral shapes across all films, confirming the presence of a single-component emissive center and the uniform dispersion of dopant molecules within the MF matrix (Fig. S16). The *Φ*_PL_ values of HICz-MF and BICz-MF were 53.12% and 83.81%, respectively (Table S4), confirming the high emission efficiencies of HICz-MF and BICz-MF. The slightly reduced *Φ*_PL_ value observed for HICz-MF may be attributed to enhanced nonradiative decay arising from the dynamic motion of the flexible cyclohexyl substituent. The afterglow quantum yields (*Φ*_Afterglow_) of HICz-MF and BICz-MF were 14.07% and 17.59%, respectively, with BICz-MF exhibiting notably superior afterglow efficiency. Collectively, these results can be attributed to the introduction of a rigid π-extended framework in BICz, which not only facilitated ISC, thereby enhancing afterglow efficiency but also stabilized triplet excitons through structural rigidification, thereby prolonging the afterglow lifetime.

**Fig. 3:**
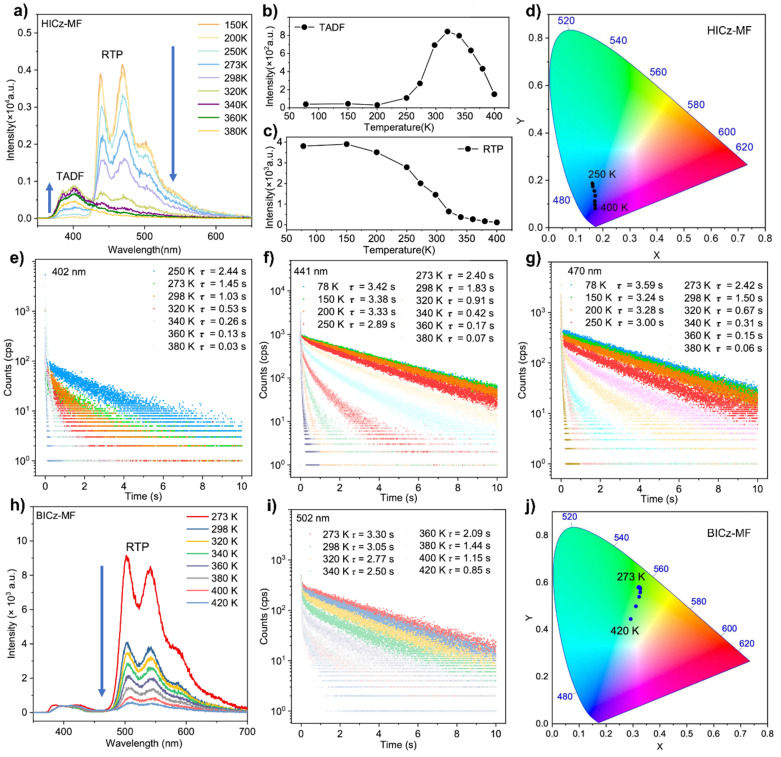
(a) Temperature-dependent delayed PL spectra of HICz-MF in a vacuum. (b) The TADF peak intensity at 402 nm at different temperatures in a vacuum. (c) The RTP peak intensity at 441 nm at different temperatures in a vacuum. (d) Temperature-dependent CIE *x*,*y* chromaticity of the HICz-MF film in vacuum (e) emission decay curves probed at 402 nm for HICz-MF at different temperatures in a vacuum. (f) Emission decay curves probed at 441 nm for HICz-MF at different temperatures in a vacuum. (g) Emission decay curves probed at 470 nm for HICz-MF at different temperatures in a vacuum. (h) Temperature-dependent delayed PL spectra for BICz-MF film in a vacuum. (i) Emission decay curves probed at 502 nm for BICz-MF at different temperatures in a vacuum. (j) Temperature-dependent CIE *x*,*y* chromaticity of BICz-MF film in a vacuum.

To reveal the nature of the delayed emission, temperature-dependent photoluminescence spectra of HICz-MF and BICz-MF films were recorded (Fig. 3). The 402 nm emission peak of the HICz-MF film was almost absent between 78 K and 250 K, but it gradually emerged upon heating to 298 K and eventually became the dominant emission at higher temperatures (Fig. 3a), indicating a thermally activated delayed fluorescence (TADF) mechanism. The intensity of the TADF emission peak increased from 273 to 320 K and then gradually diminished at higher temperatures (Fig. 3b). Correspondingly, the relative contribution of the TADF component increased significantly with temperature. As observed, the TADF emission of the HICz-MF film exhibited photophysical behavior distinct from that of typical RTP systems. This indicated that thermal activation was required for generating the deep-blue afterglow. Meanwhile, triplet–triplet annihilation (TTA) can be ruled out due to the low dopant concentration and the observation that both emission bands exhibited comparable ultralong lifetimes. Similar behavior was also observed in the ICz-MF film (Fig. S17). Therefore, the delayed fluorescence observed in HICz can be attributed to TADF emission. In parallel, the phosphorescence emission band of HICz-MF at 441 nm consistently decreased in intensity with increasing temperature (Fig. 3c). At lower temperatures (*e.g.*, 273 K), the afterglow is nearly pure phosphorescence, appearing as bright sky-blue emission. Upon heating, RTP was gradually suppressed while the TADF contribution was enhanced, ultimately leading to blue-violet afterglow above 298 K (Fig. 3d).^50^ In addition, the lifetime of the emission at 402 nm, 441 nm and 470 nm decreased with increasing temperature (Fig. 3e–g), which was consistent with the characteristic behavior of systems exhibiting both TADF and RTP. In contrast, BICz-MF did not exhibit a discernible delayed fluorescence component, confirming that the emission originated from RTP-dominated single-mode afterglow (Fig. 3h). As the temperature increased from 250 K to 400 K, both the phosphorescence intensity and delayed lifetime of BICz-MF gradually decreased (Fig. 3i), whereas the afterglow color remained unchanged (Fig. 3j). This temperature-dependent response matched the RTP characteristics, in which triplet exciton decay dominated the emission process. Compared with HICz-MF, BICz-MF film exhibited a significantly longer afterglow lifetime (3.05 s) and a higher afterglow quantum yield (17.59%), demonstrating its superior persistent luminescence performance.

**Fig. 4:**
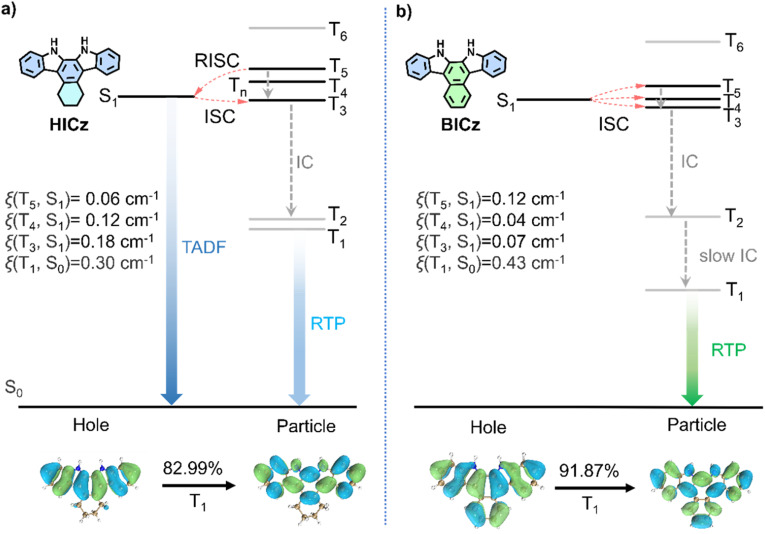
(a) Calculated energy levels, intersystem crossing (ISC) channels, and SOC constants between singlet and triplet states; and optimized molecular geometry, natural transition orbitals (NTOs) and transition probability for the T_1_ state of HICz. (b) The corresponding data for BICz.

To gain deeper theoretical insight into the distinct photophysical properties of HICz-MF and BICz-MF, density functional theory (DFT) calculations were conducted (Fig. 4). The ground-state geometries of HICz and BICz were optimized using B3LYP/6-311G (d,p), whereas the excited-state energy levels and SOC constants were evaluated at the M06-2X/6-311G (d,p) level.^51–53^ Time-dependent DFT (TD-DFT) results revealed that HICz (S_1_ → T_3_–T_5_) and BICz (S_1_ → T_3_–T_5_) both possess three feasible ISC channels. The calculated SOC constants between T_1_ and S_0_ [*ξ*(T_1_, S_0_)] slightly increased from 0.30 (HICz) to 0.43 cm^−1^ (BICz), indicating that the ISC process in BICz-MF is more efficient than in HICz-MF (Fig. 4). For HICz, the introduction of a flexible cyclohexyl substituent enhanced molecular vibration and orbital mixing, leading to a larger SOC constant and an accelerated RISC rate. The calculated *k*_RISC_ value of HICz (45.16 s^−1^) was 6.44 times higher than that of ICz, consistent with its markedly enhanced TADF quantum yield (Table S5). For BICz, theoretical calculations showed that the SOC values associated with the ISC channels were not substantially larger than those of HICz, suggesting that SOC enhancement was not the primary factor governing its photophysical behavior. Instead, the much larger energy gap in BICz seemed to play a decisive role in severely suppressing the RISC process, thereby forcing the system toward pure phosphorescence emission.^54^ In particular, BICz exhibited a larger energy gap (Δ*E*_T_3_–T_1__ = 0.99 eV) between the concerned triplet energy levels, while its T_2_ level (3.45 eV) remained similar to that of HICz (Δ*E*_T_3_–T_1__ = 0.69 eV). Although the theoretically calculated Δ*E*_T_4_–S_1__ value for BICz is extremely small (0.008 eV), which in principle strongly favors RISC, the ISC and RISC processes are collectively governed by three factors: the energy gap, spin–orbit coupling (SOC), and reorganization energy.^54–56^ Given that the SOC value for this pathway is only 0.04 cm^−1^, RISC is significantly suppressed. We recalculated the Huang–Rhys factors and total reorganization energies for the T_1_ → S_0_ transitions of HICz and BICz, and found that both the Huang–Rhys factor (0.475) and total reorganization energy (2219.26 cm^−1^) of BICz are generally larger than those of HICz (Fig. S18). According to Marcus theory, a larger reorganization energy suppresses the RISC rate, making the RISC process less competitive with phosphorescence. Consequently, BICz is less likely to exhibit TADF. Moreover, the larger total reorganization energy of BICz indicates more pronounced structural relaxation upon excitation, leading to significant energy stabilization and the formation of localized excited states. Thus, excitons in BICz first underwent internal conversion (IC) from higher triplet states to T_2_, and this was followed by a very slow IC process from T_2_ to T_1_, resulting in the prolonged phosphorescence lifetime observed in BICz-MF film. This stepwise relaxation pathway effectively trapped the excitons in the T_1_ state and substantially retarded their decay, giving rise to the ultralong phosphorescence lifetime observed in BICz-MF film.

Natural transition orbital (NTO) analysis further revealed that the triplet states (T_1_) of HICz and BICz possessed dominant (π, π*) character (Fig. 4; Tables S6 and S7). Such orbital transition patterns conformed to El-Sayed's rule, enabling a spin-allowed ISC process for the realization of efficient and long-lived RTP materials. In general, (π, π*) triplet states lie at a lower energy level and are spatially delocalized compared with (n, π*) states, which significantly enhances their intrinsic stability. The extensive conjugation within the π-framework effectively reduced vibronic coupling, thereby suppressing nonradiative decay pathways. Consequently, triplet excitons in BICz, with its more pronounced (π, π*) character, were more effectively confined within the T_1_ manifold and underwent substantially slower radiative decay. The enhanced delocalization and rigidity associated with the enlarged (π, π*) framework suppressed vibrational dissipation and nonradiative pathways, thereby extending the lifetime of the triplet population and yielding markedly prolonged RTP emission. Furthermore, the dominance of the (π, π*) configuration also reinforced host-guest interactions with the rigid MF polymer matrix. The large, planar π-systems facilitated stronger noncovalent confinement and reduced molecular motion, further diminishing the probability of vibrationally induced quenching. This synergistic stabilization was particularly pronounced in BICz, whose extended rigid π-conjugation enlarged the (π, π*) contribution, resulting in a remarkably long T_1_ lifetime and a significantly enhanced phosphorescence quantum yield. Altogether, the NTO results demonstrated that the amplified (π, π*) nature in the T_1_ state was a key factor governing the improved *Φ*_Phos_ values and ultralong lifetimes observed for the ICz derivatives, offering a clear molecular-level explanation for their exceptional afterglow performance.

**Fig. 5:**
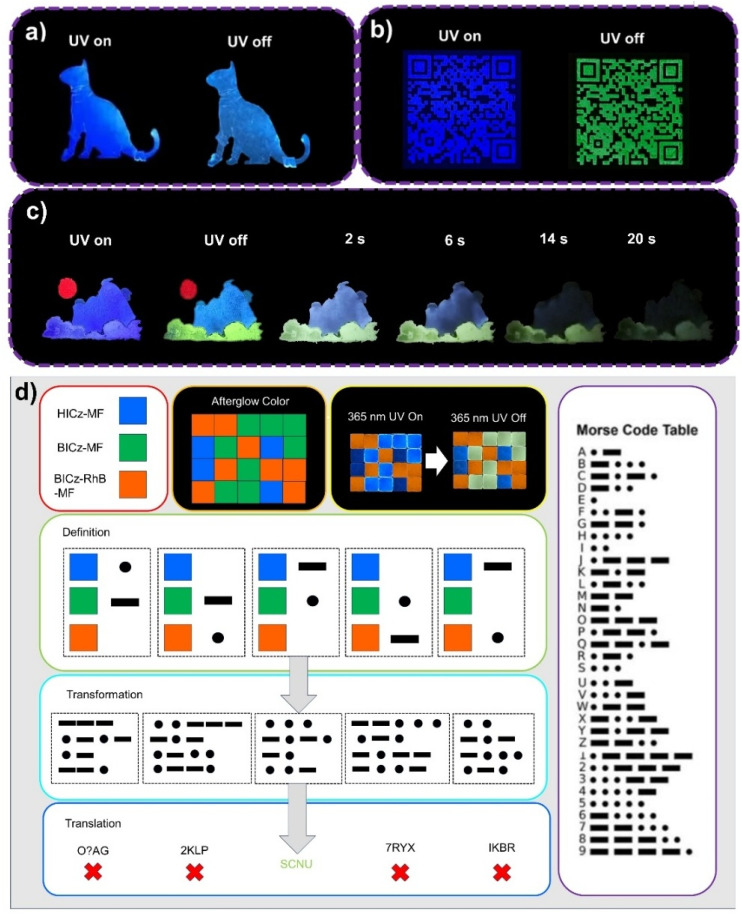
(a) A cat pattern prepared from HICz-MF. (b) A QR code encryption pattern prepared from BICz-MF. (c) A colorful pattern prepared from HICz-MF, BICz-MF and BICz-RhB-MF. (d) Information encryption realized by employing the organic afterglow colors of HICz-MF, BICz-MF and BICz-MF@RhB films to define Morse code symbols.

Due to the facile processability and excellent photophysical properties of HICz-MF and BICz-MF, they can be fabricated into large-area luminescent patterns for anti-counterfeiting applications. For example, they can be further applied to prepare multicolor luminescent patterns, such as the cat-shaped pattern shown in Fig. 5a. Furthermore, BICz-MF was used as anti-counterfeiting ink to print a phosphorescent QR code. When the QR code was scanned, the South China Normal University emblem appeared, clearly demonstrating that highly complex micro-patterns can be distinctly displayed with bright and long-lasting afterglow emission (Fig. 5b).

To achieve high-efficiency and long-lifetime multicolor afterglow emission, a triplet-sensitized Förster resonance energy transfer (TS-FRET) strategy was employed, using Rhodamine B (RhB) as the guest emitter and BICz-MF as the energy donor.^34^ By co-doping the organic luminophores and RhB dye at various mass ratios, an increase in the emissive peak was observed upon increasing the RhB concentration (Fig. S19), accompanied by long-lived red afterglow emission on the millisecond-to-second scale, confirming efficient energy transfer. Considering both lifetime and quantum yield (Table S8), the film with an optimal mass ratio of 1 : 1 (BICz : RhB) yielded an afterglow lifetime of 0.83 s and a quantum yield of 12.28%, which ranked among the best values reported for polymer-based RTP systems. These materials, HICz-MF, BICz-MF and BICz-RhB-MF, were further employed to fabricate large-area multicolor luminescent patterns (Fig. 5c) and encrypted information displays (Fig. 5d). By freely combining thin films with different emission properties, information could be optically encoded, enriching the diversity of encryption strategies. A particularly secure approach involved defining the multicolor afterglow emission of MF films as Morse code symbols, where the correct decoding sequence revealed hidden information. As shown in Fig. 5d, a matrix composed of HICz-MF (blue), BICz-MF (yellow), and BICz-RhB-MF (red) films was used to encode the message “SCNU”. To decipher it correctly, one must assign the afterglow colors to specific Morse code symbols. For example, blue, yellow, and red correspond, respectively, to “-”, “⋅”, and “blank” (interference) signals. According to the Morse code table, “⋅⋅⋅” represents S, “-⋅-⋅” represents C, “-⋅” represents N, and “⋅⋅-” represents U, constructing the message “SCNU.” In contrast, an incorrect color-to-symbol assignment produced false information, which highlighted the robustness and complexity of this encryption method.

## Conclusions

In this work, two derivatives, HICz bearing a flexible cyclohexyl group and BICz incorporating a rigid phenyl π-extension, were synthesized and then embedded into a melamine-formaldehyde (MF) matrix. The theoretical and experimental results show that introducing six-membered ring substituents provides an effective strategy to modulate π-conjugation and molecular flexibility, which are related to the SOC intensity, Δ*E*_ST_, and the ISC process, thereby enabling tuning of the competition between TADF and RTP pathways in HICz-MF and BICz-MF materials. Although the flexible cyclohexyl unit in HICz promotes RISC-favorable conformations, its intrinsic mobility simultaneously enhances nonradiative decay, limiting the phosphorescence lifetime (1.61 s) and *Φ*_PL_ (53.51%). In contrast, rigid π-extension in BICz fundamentally alters the excited-state landscape. Comprehensive photophysical studies and DFT calculations reveal that the rigid framework not only promotes ISC but also significantly enlarges Δ*E*_ST_, thereby suppressing RISC and stabilizing the triplet manifold. Consequently, BICz-MF exhibits purely RTP-dominated emission with an ultralong lifetime of 3.05 s and a high phosphorescence quantum yield of 17.59%. These results establish rigid framework engineering as a powerful and generalizable design principle for constructing efficient, long-lived polymer-based RTP materials. Moreover, the integration of a triplet-sensitized Förster resonance energy transfer (TS-FRET) strategy enables multicolor afterglow emission, supporting applications in optical encryption, anti-counterfeiting, and hierarchical information storage. Overall, this study demonstrates that rational rigid-flexible structural modulation offers a robust strategy for tailoring ISC dynamics and unlocking the full potential of ICz-based organic afterglow systems.

## Author contributions

Yu Lang: writing – original draft, conceptualization, data curation and formal analysis. Jiahui Sun: data curation, formal analysis and investigation. Mengyao Jiang: formal analysis and data curation. Qingyun Jiang: investigation. Yongkang Du: investigation. Feng Wang: funding acquisition and investigation. Jiadong Zhou: writing – review & editing, and formal analysis. Guang Shi: funding acquisition and conceptualization. Bingjia Xu: supervision and methodology. Cong Liu: writing – review & editing, and supervision.

## Conflicts of interest

There are no conflicts to declare.

## Supplementary Material

SC-017-D5SC10031C-s001

## Data Availability

Data will be made available on request from Cong Liu. Supplementary information (SI): the experimental procedures, theoretical calculations, characterization results of ^1^H NMR, ^13^C NMR and high-resolution mass spectra, and supplemental spectra. See DOI: https://doi.org/10.1039/d5sc10031c.

## References

[cit1] Yang X., Waterhouse G. I. N., Lu S. Y., Yu J. H. (2023). Recent Advances in the Design of Afterglow Materials: Mechanisms, Structural Regulation Strategies and Applications. Chem. Soc. Rev..

[cit2] Song T. H., Liu H. L., Ren J., Wang Z. W. (2024). Achieving TADF and RTP with Stimulus-Responsiveness and Tunability from Phenothiazine-Based Donor-Acceptor Molecules. Adv. Opt. Mater..

[cit3] Gao R., Kodaimati M. S., Yan D. (2021). Recent Advances in Persistent Luminescence Based on Molecular Hybrid Materials. Chem. Soc. Rev..

[cit4] Gmelch M., Thomas H., Fries F., Reineke S. (2019). Programmable Transparent Organic Luminescent Tags. Sci. Adv..

[cit5] Abdollahi A., Roghani-Mamaqani H., Razavi B., Salami-Kalajahi M. (2020). Photoluminescent and Chromic Nanomaterials for Anticounterfeiting Technologies: Recent Advances and Future Challenges. ACS Nano.

[cit6] Chen C., Gao H., Ou H., Kwok R. T. K., Tang Y., Zheng D., Ding D. (2022). Amplification of Activated Near-Infrared Afterglow Luminescence by Introducing Twisted Molecular Geometry for Understanding Neutrophil-Involved Diseases. J. Am. Chem. Soc..

[cit7] Liu X. W., Zhao W., Wu Y., Meng Z., He Z., Qi X., Ren Y., Yu Z., Tang B. (2022). Photo-Thermo-Induced Room-Temperature Phosphorescence through Solid-State Molecular Motion. Nat. Commun..

[cit8] Dai W., Zhang Y., Wu X., Guo S., Ma J., Shi J., Tong B., Cai Z., Xie H., Dong Y. (2022). Red-Emissive Organic Room-Temperature Phosphorescence Material for Time-Resolved Luminescence Bioimaging. CCS Chem..

[cit9] Su Y., Zhang Y., Wang Z., Gao W., Jia P., Zhang D., Yang C., Li Y., Zhao Y. (2020). Excitation-Dependent Long-Life Luminescent Polymeric Systems under Ambient Conditions. Angew. Chem., Int. Ed..

[cit10] Wei J., Xiao Y., Luo J., He Z., Chen J., Peng Q., Kuang D. (2025). Anion-π Interaction Guided Switchable TADF and Low-Temperature Phosphorescence in Phosphonium Salts for Multiplexed Anti-Counterfeiting. Chem. Sci..

[cit11] Yuan W., Shen X., Zhao H., Lam J. W. Y., Tang L., Lu P., Wang C., Liu Y., Wang Z., Zheng Q., Sun J., Ma Y., Tang B. (2010). Crystallization-Induced Phosphorescence of Pure Organic Luminogens at Room Temperature. J. Phys. Chem. C.

[cit12] Shi H., Yao W., Ye W., Ma H., Huang W., An Z. (2022). Ultralong Organic Phosphorescence: From Material Design to Applications. Accounts Chem. Res..

[cit13] Chen K. C., Liu B. (2019). Enhancing the Performance of Pure Organic Room-Temperature Phosphorescent Luminophores. Nat. Commun..

[cit14] Liu Q., Qian J., Xia S., Liu M., Chai Y., Dai W., Lei Y., Wu H., Huang X. (2025). Constructing Organic Doped Ultra-Long Afterglow Materials with High Temperature Resistance and Wide Color Gamut through Förster-Resonance Energy Transfer. Chem. Eng. J..

[cit15] Wang Y., Huang H., Liu Q., Liu M., Dai W., Lei Y., Wang X., Huang X., Wu H. (2024). Organic Doped Red Room-Temperature Afterglow Materials Based on 2,3,5-Triarylfuro[3,2-B]Pyridines through Förster-Resonance Energy Transfer. Chem. Eng. J..

[cit16] Garain S., Ansari S. N., Kongasseri A. A., Chandra Garain B., Pati S. K., George S. J. (2022). Room Temperature Charge-Transfer Phosphorescence from Organic Donor–Acceptor Co-Crystals. Chem. Sci..

[cit17] Xia Y., Zhu C., Cao F., Shen Y., Ouyang M., Zhang Y. (2023). Host-Guest Doping in Flexible Organic Crystals for Room-Temperature Phosphorescence. Angew. Chem., Int. Ed..

[cit18] Yan X., Peng H., Xiang Y., Wang J., Yu L., Tao Y., Li H., Huang W., Chen R. (2022). Recent Advances on Host-Guest Material Systems toward Organic Room Temperature Phosphorescence. Small.

[cit19] Wang J., Lou X., Wang Y., Tang J., Yang Y. (2021). Recent Advances of Polymer-Based Pure Organic Room Temperature Phosphorescent Materials. Macromol. Rapid Commun..

[cit20] Guo S., Dai W., Chen X., Lei Y., Shi J., Tong B., Cai Z., Dong Y. (2021). Recent Progress in Pure Organic Room Temperature Phosphorescence of Small Molecular Host-Guest Systems. ACS Mater. Lett..

[cit21] Chen Z., Shi J., Liang G. (2025). Dual-Mode Hour-Long Afterglow in Flexible and Transparent Polymeric Materials. Chem. Eng. J..

[cit22] Wang L., Chen W., Zhuang Y., Yuan Y., Chen Y. (2025). Ionic Liquid-Assisted Fabrication of Naphthalimide-Doped Degradable Polylactide Films Exhibiting Atmospheric and Aquatic Dual-Environment Ultra-Long Afterglow. Chem. Eng. J..

[cit23] Li J., Wei K., Wu J., Wang Y., Liu S., Ma Y., Zhao Q. (2024). Simultaneously Enhancing Organic Phosphorescence Quantum Yields and Lifetimes for Triphenylphosphine Salt Doped Polymer Films. Chem. Sci..

[cit24] Ma X., Liu Y. (2021). Supramolecular Purely Organic Room-Temperature Phosphorescence. Accounts Chem. Res..

[cit25] Ma X., Wang J., Tian H. (2019). Assembling-Induced Emission: An Efficient Approach for Amorphous Metal-Free Organic Emitting Materials with Room-Temperature Phosphorescence. Accounts Chem. Res..

[cit26] Zhou W., Lin W., Chen Y., Liu Y. (2022). Supramolecular Assembly Confined Purely Organic Room Temperature Phosphorescence and Its Biological Imaging. Chem. Sci..

[cit27] Ma H., Fu L., Yao X., Jiang X., Lv K., Ma Q., Shi H., An Z., Huang W. (2024). Boosting Organic Phosphorescence in Adaptive Host-Guest Materials by Hyperconjugation. Nat. Commun..

[cit28] Chen Q. G., Qu L., Hou H., Huang J., Li C., Zhu Y., Wang Y., Chen X., Zhou Q., Yang Y., Yang C. (2024). Long Lifetimes White Afterglow in Slightly Crosslinked Polymer Systems. Nat. Commun..

[cit29] Gan N., Zou X., Qian Z., Lv A., Wang L., Ma H., Qian H., Gu L., An Z., Huang W. (2024). Stretchable Phosphorescent Polymers by Multiphase Engineering. Nat. Commun..

[cit30] Nidhankar A. D., Goudappagouda, Wakchaure V. C., Babu S. S. (2021). Efficient Metal-Free Organic Room Temperature Phosphors. Chem.
Sci..

[cit31] Chen J., Yu T., Ubba E., Xie Z., Yang Z., Zhang Y., Liu S., Xu J., Aldred M. P., Chi Z. (2019). Achieving Dual-Emissive and Time-Dependent Evolutive Organic Afterglow by Bridging Molecules with Weak Intermolecular Hydrogen Bonding. Adv. Opt. Mater..

[cit32] Wang J., Fang Y., Li C., Niu L., Fang W., Cui G., Yang Q. (2020). Time-Dependent Afterglow Color in a Single-Component Organic Molecular Crystal. Angew. Chem., Int. Ed..

[cit33] Yao X., Ma H., Wang X., Wang H., Wang Q., Zou X., Song Z., Jia W., Li Y., Mao Y., Singh M., Ye W., Liang J., Zhang Y., Liu Z., He Y., Li J., Zhou Z., Zhao Z., Zhang Y., Niu G., Yin C., Zhang S., Shi H., Huang W., An Z. (2022). Ultralong Organic Phosphorescence from Isolated Molecules with Repulsive Interactions for Multifunctional Applications. Nat. Commun..

[cit34] Wang D., Gong J., Xiong Y., Wu H., Zhao Z., Wang D., Tang B. (2023). Achieving Color-Tunable and Time-Dependent Organic Long Persistent Luminescence via Phosphorescence Energy Transfer for Advanced Anti-Counterfeiting. Adv. Funct. Mater..

[cit35] Yu J., Sun Z., Ma H., Wang C., Huang W., He Z., Wu W., Hu H., Zhao W., Zhu W. (2023). Efficient Visible-Light-Activated Ultra-Long Room-Temperature Phosphorescence Triggered by Multi-Esterification. Angew. Chem., Int. Ed..

[cit36] Zhen J., Long J., Guo X., Wang Q., Zeng X. (2024). Tryptophan-Doped Poly(vinyl alcohol) Films with Ultralong-Lifetime Room-Temperature Phosphorescence and Color-Tunable Afterglow Under Ambient Conditions. Chem. Eur. J.

[cit37] Liu R., Liu C., Fu C., Zhu Z., Chen K., Li C., Wang L., Huang Y., Lu Z. (2024). Ambient Phosphor with High Efficiency and Long Lifetime in Poly(Methyl Methacrylate) Through Charge-Transfer-Mediated Triplet Exciton Formation for Photolithography Applications. Angew. Chem., Int. Ed..

[cit38] Guan Z., Tang Z., Yao Z., Guo Q., Zhang S., Lv Z., Zhang X., Ma N., Liu X., Hu Z. (2025). “Rigid-Flexible” Strategy Realizes Robust Ultralong Phosphorescence for Multifunctional Display Unit and Photoreceptor Synapse. Adv. Mater..

[cit39] Liang Y., Hu P., Zhang H., Yang Q., Wei H., Chen R., Yu J., Liu C., Wang Y., Luo S., Shi G., Chi Z., Xu B. (2024). Enabling Highly Robust Full-Color Ultralong Room-Temperature Phosphorescence and Stable White Organic Afterglow from Polycyclic Aromatic Hydrocarbons. Angew. Chem., Int. Ed..

[cit40] Zhang H., Wu S., Liang Y., Zhang Z., Wei H., Yang Q., Hu P., Liu C., Yang Z., Zheng C., Shi G., Chi Z., Xu B. (2024). Enabling Efficient and Ultralong Room-Temperature Phosphorescence from Organic Luminogens by Locking the Molecular Conformation in Polymer Matrix. Chem. Eng. J..

[cit41] Yan R., Ding B., Li T., Yin C., Ma X. (2025). Multichannel Afterglow Regulated by Protonation-Induced Reversed Intersystem Crossing. Chem. Comm..

[cit42] Shen Y., An Z., Liu H., Yang B., Zhang Y. (2023). Excitation-Dependent Multicolour Luminescence of Organic Materials: Internal Mechanism and Potential Applications. Angew. Chem., Int. Ed..

[cit43] Sun H., Li X., Hsu C., Hung C., Wu B., Su Z., Baryshnikov G. V., Li C., Ågren H., Zhang Z., Huang W., Wu D., Chou P., Zhu L. (2025). Sulfur Lone Pairs Open Avenues for π* → n Orange-to-Red TADF and OLEDs. J. Am. Chem. Soc..

[cit44] Wang J., Niu Y., Jiang Y., Chen Z., Yao C., Yao W., He M., Zhang J. (2025). Modulating the Photophysical Properties of Isomeric Thermally Activated Delayed Fluorescence Emitters through Precise Control of Intra/Intermolecular Hydrogen Bonding for Nondoped OLEDs. Mater. Today Chem..

[cit45] Wang J., Yang Y., Gu F., Zhai X., Yao C., Zhang J., Jiang C., Xi X. (2023). Molecular Engineering Modulating the Singlet-Triplet Energy Splitting of Indolocarbazole-Based TADF Emitters Exhibiting AIE Properties for Nondoped Blue OLEDs with EQE of Nearly 20. ACS Appl. Mater. Interfaces.

[cit46] Xu L., Wei H., Xie G., Xu B., Zhao J. (2024). Ultralong MRTADF and Room-Temperature Phosphorescence Enabled Color-Tunable and High-Temperature Dual-Mode Organic Afterglow from Indolo[3,2-b]carbazole. Adv. Funct. Mater..

[cit47] Yang Y., Liang Y., Zheng Y., Li J.-A., Wu S., Zhang H., Huang T., Luo S., Liu C., Shi G., Sun F., Chi Z., Xu B. (2022). Efficient and Color-Tunable Dual-Mode Afterglow from Large-Area and Flexible Polymer-Based Transparent Films for Anti-Counterfeiting and Information Encryption. Angew. Chem., Int. Ed..

[cit48] Chen M., Liu B., Ren J., Zhang C., Ren Z., Guan Z. (2025). Stretchable, Ultralong Room-Temperature Phosphorescence Poly(urethane-urea) Elastomer Resistant to Humidity and Heat. Adv. Mater..

[cit49] Jian M., Song Z., Chen X., Zhao J., Xu B., Chi Z. (2022). Afterglows from the Indolocarbazole Families. Chem. Eng. J..

[cit50] Xu B., Song Z., Zhang M., Zhang Q., Jiang L., Xu C., Zhong L., Su C., Ban Q., Liu C., Sun F., Zhang Y., Chi Z., Zhao Z., Shi G. (2021). Controlling the Thermally Activated Delayed Fluorescence of Axially Chiral Organic Emitters and Their Racemate for Information Encryption. Chem. Sci..

[cit51] Frisch M. J. , Trucks G. W., Schlegel H. B., Scuseria G. E., Robb M. A., Cheeseman J. R., Scalmani G., Barone V., Petersson G. A., Nakatsuji H., Li X., Caricato M., Marenich A. V., Bloino J., Janesko B. G., Gomperts R., Mennucci B., Hratchian H. P., Ortiz J. V., Izmaylov A. F., Sonnenberg J. L., Williams-Young D., Ding F., Lipparini F., Egidi F., Goings J., Peng B., Petrone A., Henderson T., Ranasinghe D., Zakrzewski V. G., Gao J., Rega N., Zheng G., Liang W., Hada M., Ehara M., Toyota K., Fukuda R., Hasegawa J., Ishida M., Nakajima T., Honda Y., Kitao O., Nakai H., Vreven T., Throssell K., Montgomery J. A., Peralta J. E., Ogliaro F., Bearpark M. J., Heyd J. J., Brothers E. N., Kudin K. N., Staroverov V. N., Keith T. A., Kobayashi R., Normand J., Raghavachari K., Rendell A. P., Burant J. C., Iyengar S. S., Tomasi J., Cossi M., Millam J. M., Klene M., Adamo C., Cammi R., Ochterski J. W., Martin R. L., Morokuma K., Farkas O., Foresman J. B. and Fox D. J., Gaussian 16 Rev. C.01, 2016

[cit52] Lu T., Chen F. (2012). Multiwfn: A multifunctional wavefunction analyzer. J. Comput. Chem..

[cit53] Humphrey W. F., Dalke A., Schulten K. (1996). VMD: Visual Molecular Dynamics. J. Mol. Graph..

[cit54] Peng Q., Niu Y., Shi Q., Gao X., Shuai Z. (2013). Correlation Function Formalism for Triplet Excited State Decay: Combined Spin-Orbit and Nonadiabatic Couplings. J. Chem. Theory Comput..

[cit55] Peng Q., Fan D., Duan R., Yi Y., Niu Y., Wang D., Shuai Z. (2017). Theoretical Study of Conversion and Decay Processes of Excited Triplet and Singlet States in a Thermally Activated Delayed Fluorescence Molecule. J. Phys. Chem. C.

[cit56] Shuai Z., Peng Q. (2014). Excited States Structure and Processes: Understanding Organic Light-Emitting Diodes at the Molecular Level. Phys. Rep..

