# Supplementary material for: Efficient persistent afterglow modulation using extended Indolo[2,3-a]carbazoles with six-membered rings in a polymer matrix
Source: Chem Sci. 2026 Mar 6;17(18):9098–107. doi: 10.1039/d5sc10031c (PMC12993868; doi:10.1039/d5sc10031c)
Supplement: SC-017-D5SC10031C-s001 [file SC-017-D5SC10031C-s001.pdf]

Supplementary Information (SI) for Chemical Science.
This journal is © The Royal Society of Chemistry 2026

Supporting Information for

Efficient Persistent Afterglow Modulation for Extending Indolo[2,3-a]carbazole with Six-Membered Ring in Polymer Matrix

Yu Lang[a], Jiahui Sun[a], Mengyao Jiang[a], Qingyun Jiang[a], Yongkang Du[a], Feng Wang[b,c], Jiadong Zhou[a,*], Guang Shi[a,*], Bingjia Xu[d], Cong Liu[a,*]

[a] *School of Chemistry, South China Normal University, Guangzhou 510006, PR China.*
[b] *Kingfa Science & Technology Co., Ltd., Guangzhou 510663, China.*
[c] *Zhuhai Vanteque Specialty Engineering Plastics Co., Ltd., Zhuhai 519050, China.*
[d] *School of Environmental and Chemical Engineering, Wuyi University, Jiangmen 529020, PR China.*

Contents

1. Experimental section 3

1.1 Chemical reagents and materials 3

1.2 Instruments and measurements 3

1.3 Theoretical calculations.. 3

1.4 Synthesis 4

1.5 Preparation of melamine-formaldehyde (MF) polymer film.................................. 6

2. Typical luminescent materials with organic afterglow properties.............................. 7

3. ^{1}H NMR, ^{13}C NMR, and high-resolution mass spectra 10

4. Photophysical properties of ICz, HICz, and BICz... 15

1. Experimental section

1.1 Chemical reagents and materials

11,12-Dihydroindolo[2,3-a]carbazole, indole, 2-chlorocyclohexanone, 2,3-diamino- thalene, 2-bromoiodobenzene, copper(II) sulfate pentahydrate, palladium(II) acetate, (oxybis(2,1-phenylene))bis(diphenylphosphine)[DPEPhos], potassium tert-butoxide, potassium carbonate, tricyclohexylphosphonium tetrafluoro-borate and melamine were all purchased from Bidepharm. These chemical reagents were used as received. Other reagents and organic solvents were purchased from Energy Chemical and Guangzhou Chemical Reagent Factory with analytical grade and used without further purification.

1.2 Instruments and measurements

^{1}H and ^{13}C nuclear magnetic resonance spectra of the intermediates and the final products were obtained on a Bruker AVANCE spectrometer (600 MHz) by employing $CDCl_3$ and DMSO-d_6 as solvents and tetramethylsilane as internal standard. In addition, steady-state PL spectra, delayed emission spectra, absolute PL quantum yields, and time-resolved emission decay curves were collected by a spectrometer (FLS980) equipped with a calibrated integrating sphere and a thermostat (Oxford) from Edinburgh Instruments. TG curves were recorded by a thermogravimetric analyzer (TG 209 F3, Germany) at a heating rate of 10 °C/min under nitrogen atmosphere. The UV-vis absorption spectroscopy (UV-Vis) were collected on a U-3900 UV spectrophotometer (Hitachi, Japan). The delayed emission spectra at 77 K were collected on an Ocean Optics spectrophotometer equipped with a QE65 Pro charge-couple device (CCD) detector.

1.3 Theoretical calculations

The molecular geometries of the indolocarbazole and its derivatives without and with MF polymer fragment at ground state were optimized using the density functional theory (DFT) method at the B3LYP/6-311g(d,p) level in the Gaussian 16 program. Their singlet (S_n) and triplet (T_n) energy levels were estimated through a combination of TD-DFT at the 6-311g (d, p) level. Spin-orbit coupling (SOC) matrix elements between the singlet and triplet excited states were calculated with spin-orbit mean-field (SOMF) methods. Natural transition orbital (NTO) analysis was also performed, and the results were extracted by using the software of Multiwfn (version 3.8) and then visualized via the VMD software.

The chemical structures of the selected MF fragment are as follows:

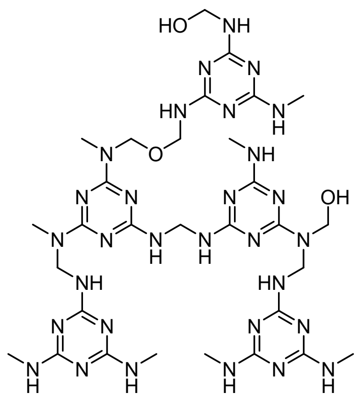

1.4 Synthesis

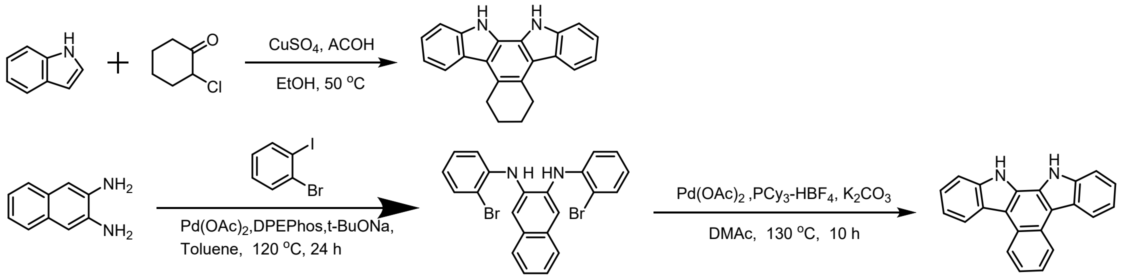

Scheme S1. The synthetic routes of HICz and BICz.

11,12-Dihydroindolo[2,3-a]carbazole (ICz):

ICz was purchased from a reagent company and purified by silica gel column chromatography with dichloromethane/petroleum ether (*V*/*V*=2:1) as eluent. ^{1}H NMR (600 MHz, DMSO-d6) δ 11.05 (s, 2H), 8.15 (m, J = 7.9, 0.9 Hz, 2H), 7.91 (s, 2H), 7.69 (m, J = 8.0, 0.9 Hz, 2H), 7.39 (m, J = 8.1, 7.0, 1.2 Hz, 2H), 7.20 (m, J = 7.9, 7.0, 1.0 Hz, 2H). ^{13}C NMR (151 MHz, DMSO-d_6) δ 139.43, 126.09, 124.96, 124.23, 120.54, 120.16, 119.36, 112.05, 40.53, 40.40. ESI-MS: m/z $[M+H]^+$ calculated for $C_{18}H_{13}N_2^+$: 257.1075; found: 257.1073.

5,6,7,8,13,14-hexahydrobenzo[c]indolo[2,3-a]carbazole (HICz):

2-Chlorocyclohexanone (0.69 g, 5.17 mmol) and acetic acid glacial (0.98 g, 16.32 mmol) were dissolved in ethanol (20 mL). Then copper sulfate pentahydrate (1.23 g, 4.91 mmol) and indole (0.97 g, 8.25 mmol) were added after stirring for 30 minutes under an argon atmosphere. The reaction mixture was stirred at 50 °C until indole is completely consumed (monitored by TLC). After cooling to room temperature, the mixture was filtered, and the solvent of the filtrate was removed by rotary evaporation. The crude product was then separated and purified by silica gel column

chromatography with ethyl acetate and petroleum ether (*V*/*V*=1:4) as eluent. The resulting solid was further recrystallized twice from dichloromethane/methanol to give a white powder (0.75 g, yield 58.59 %). ^{1}H NMR (600 MHz, DMSO-d_6, TMS) δ: 11.01 (s, 2H), 8.18 (d, J = 7.9 Hz, 2H), 7.71-7.66 (m, 2H), 7.40-7.34 (m, 2H), 7.22-7.16 (m, 2H), 3.39 (p, J = 3.2 Hz, 4H), 2.04 (p, J = 3.0 Hz, 4H). ^{13}C NMR (151 MHz, Chloroform-d) δ 138.90, 137.59, 132.25, 129.25, 129.05, 127.17, 125.72, 125.33, 125.15, 124.85, 124.74, 124.48, 124.44, 124.27, 123.99, 123.52, 122.17, 120.99, 114.88, 111.56. ESI-MS: m/z $[M^{+}H]^{+}$ calculated for $C_{22}H_{19}N_2^{+}$: 311.1544; found: 311.1543.

N^2,N^3-bis(2-bromophenyl)naphthalene-2,3-diamine:

2,3-diaminonaphthalene (0.50 g, 3.17 mmol), 2-bromoiodobenzene (3.12 g, 11.02 mmol) and potassium tert-butoxide (0.65 g, 6.77 mmol) were dissolved in toluene (20 mL) and bubbled for 30 minutes. Then, bis (2-diphenylphosphophenyl) ether (0.09 g, 0.17 mmol) and palladium acetate (0.08 g, 0.37 mmol) were added to the reaction solution. The mixture was stirred at 120 °C for 24 hours. After cooling to room temperature, the mixture was filtered and the solvent in the filtrate was removed by rotary evaporation. The crude product was then separated and purified by silica gel column chromatography with dichloromethane and petroleum ether (*V*/*V*=1:4) as eluent. The resulting solid was further purified by twice recrystallizing from a dichloromethane/methanol mixture, affording the product as a white powder (0.71 g, yield 43.53%). ESI-MS: m/z $[M+H]^{+}$ calculated for $C_{22}H_{16}Br_2N_2^{+}$: 468.9727; found: 468.9733.

13,14-dihydrobenzo[c]indolo[2,3-a]carbazole (BICz):

N^2,N^3-bis(2-bromophenyl)naphthalene-2,3-diamine (0.70 g, 1.50 mmol) and 2-potassium carbonate (0.66 g, 4.78 mmol) were dissolved in N,N-dimethylacetamide (20 mL) and bubbled for 30 minutes. Then, DPEPhos (0.08 g, 0.22 mmol) and palladium acetate (0.04 g, 0.19 mmol) were added to the reaction solution. The mixture was stirred at 130 °C for 10 hours. After cooling to room temperature, the mixture was filtered and the solvent in the filtrate was removed by rotary evaporation. The crude product was then separated and purified by silica gel column chromatography with dichloromethane and petroleum ether (*V*/*V*=1:1) as eluent. The resulting solid was further purified by twice recrystallizing from a dichloromethane/methanol mixture, affording the product as a white powder (0.12 g, yield 26.14%). ^{1}H NMR (600 MHz, DMSO-d6) δ 11.57 (s, 2H), 8.92 (m, J = 7.0, 3.5 Hz, 2H), 8.64 (d, J = 8.0 Hz, 2H), 7.85 (d, J = 8.0 Hz, 2H), 7.67 (m, J = 6.7, 3.3 Hz, 2H), 7.50-7.42 (m, 2H), 7.39-7.33 (m, 2H). ^{13}C NMR (151

MHz, DMSO-d_6) δ 138.89, 126.94, 126.42, 124.45, 124.25, 124.09, 124.08, 121.76, 120.62, 113.71, 112.69, 40.54, 40.40.ESI-MS: m/z $[M+H]^+$ calculated for $C_{22}H_{14}N_2^+$: 307.12277; found: 307.12297.

1.5 Preparation of melamine-formaldehyde (MF) polymer film

Formaldehyde aqueous solution (37%, 20.00 g) was added into a 100 mL beaker. Then melamine (12.60 g) was added after the pH value was adjusted to 8.5 with triethanolamine. After sealing with plastic wrap, the solution was heated to 130 °C and stirred for an hour to obtain a clear and transparent MF prepolymer solution. ICz and its derivatives in THF (2.00 mg/mL; 50 μL, 250 μL, 500 μL and 2500 μL corresponding to doping concentrations of 0.01%, 0.05%, 0.10% and 0.50% respectively) were mixed with melamine resin prepolymer solution (60%, 1 g). The resulting mixtures were treated in an ultrasonic bath for about 30 minutes, followed by drop-casting on quartz plates. After drying at 150 °C for 10 minutes, the MF polymer was scraped off and pressed into a translucent MF polymer film at 160 °C.

2. Typical luminescent materials with organic afterglow properties

Table S1. Typical polymer-based organic afterglow materials with $\Phi_{phos.}$ > 15% and $\tau_{phos.}$ > 1 s under ambient conditions from 2021 to the present.[1-8]

**1-1**<br>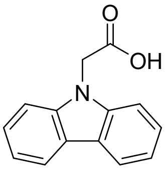<br>Doped in PVA	**1-2**<br>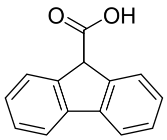<br>Doped in PVA	**2**<br>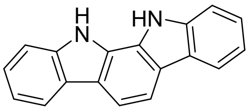<br>Doped in PVA
**3-1**<br>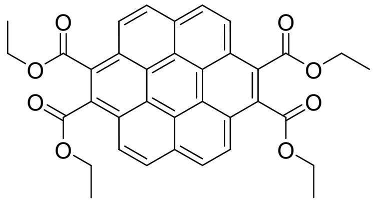<br>Doped in PVA	**3-2**<br>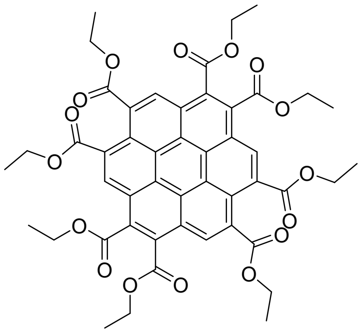<br>Doped in PVA	
**4-1**<br>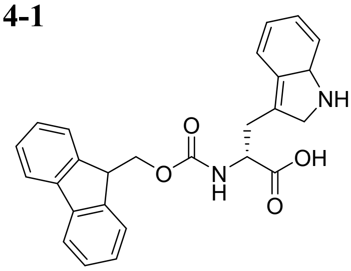<br>Doped in PVA	**4-2**<br>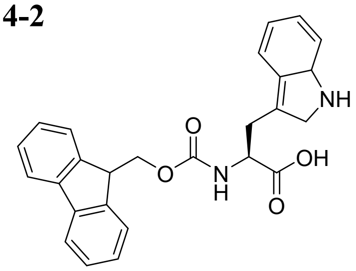<br>Doped in PVA	**5**<br>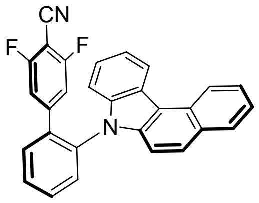<br>Doped in PMMA
**6**<br>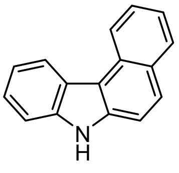<br>Doped in PVA	**7**<br>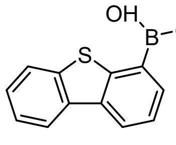<br>Doped in PVA	**8**<br>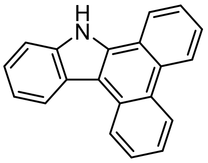<br>Doped in PVP
**9**<br>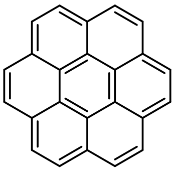<br>Doped in MF	**10**<br>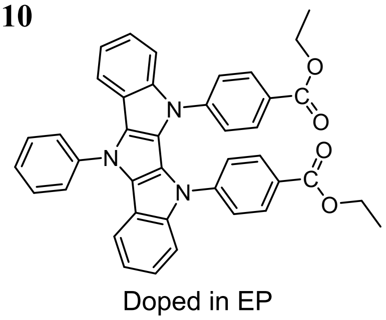<br>Doped in EP	**11**<br>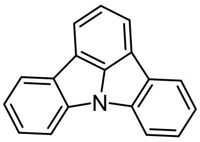<br>Doped in MF

Table S2. Photophysical properties of the typical polymer-based organic afterglow materials with $\Phi_{phos.}$> 15% and $\tau_{phos.}$ > 1 s under ambient conditions from 2021 to the present.

System	$\Phi_{phos.}$ [%]	$\tau_{phos.}$ [ms]	Ref.
1-1	20.10	2730	*Nat. Commun.* **2022**, 13, 4890
1-2	50.00	3210	*Nat. Commun.* **2022**, 13, 4890
2	44.1	2040	*Adv. Funct. Mater*. **2023**, 33, 2208895
3-1	36.5	2030	*Angew. Chem. Int. Ed*. **2023**, 62, e202316647
3-2	25.2	1980	*Angew. Chem. Int. Ed*. **2023**, 62, e202316647
4-1	39.67	3829.85	*Chem. Eur. J.* **2024**, 30, e202304137
4-2	48.30	3859.70	*Chem. Eur. J.* **2024**, 30, e202304137
5	30	1920	*Angew. Chem. Int. Ed*. **2024**, 63, e202312534
6	46.34	2090.39	*Adv. opt. Mater*. **2024**, 12, 2301812
7	25.35	1320	*Adv. opt. Mater*. **2024**, 12, 2302424
8	42.7	4710	*Adv. Mater.* **2025**, 37, no. 41 (2025): e07192
9	22.7	4830	*Angew. Chem. Int. Ed*. **2024**, 63, e202318516
10	24.35	2030	*Adv. opt. Mater.* **2024**, *12*, 2400642
11	38.31	2726.81	*Chem. Eng. J.* **2024**, 497, 154949
BICz	17.59	3060	**This work**
HICz	14.07	1620	

Table S3. Luminescent materials with organic afterglow from ICz and its derivatives. [2, 9-11]

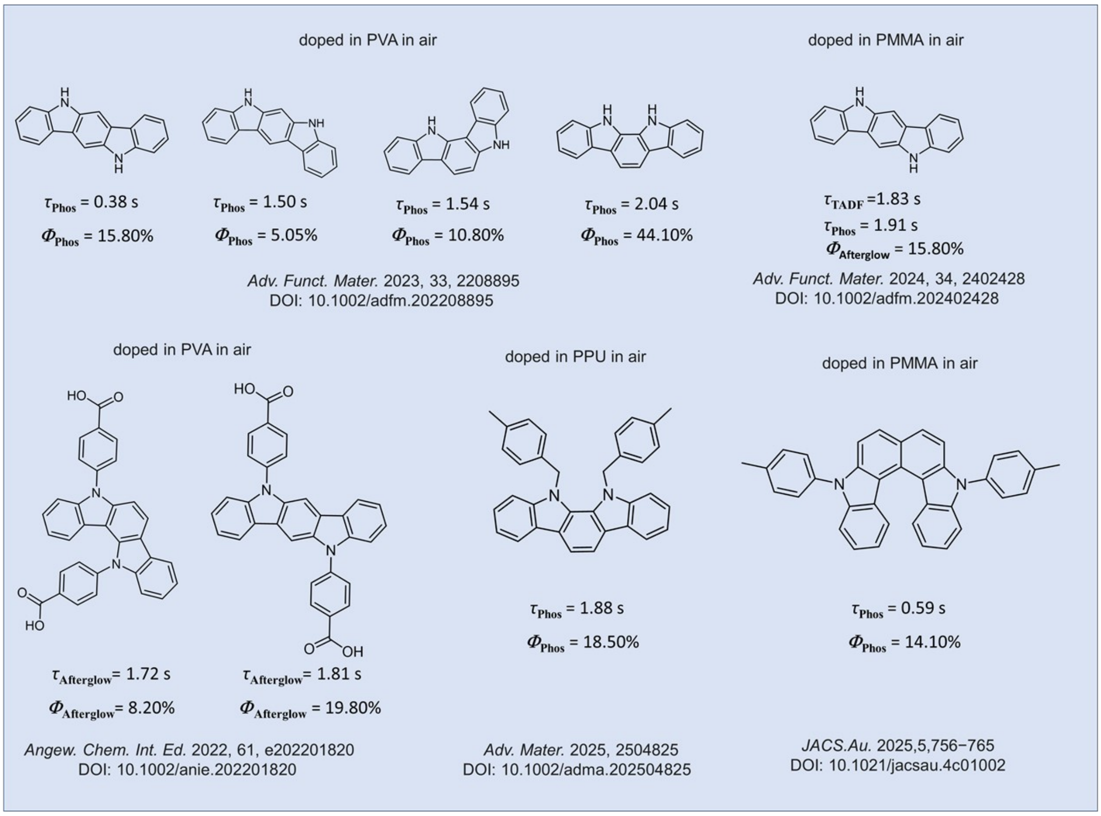

3. ^{1}H NMR, ^{13}C NMR, and high-resolution mass spectra

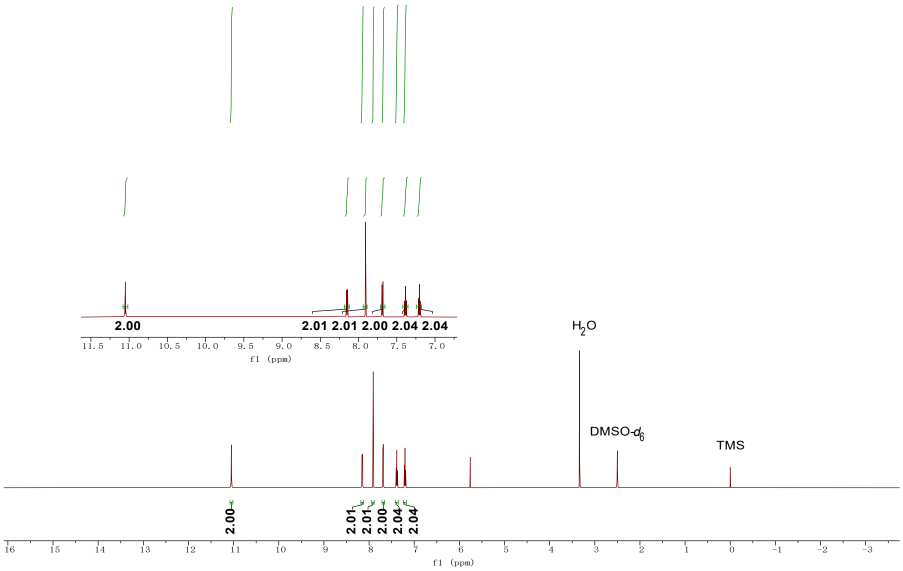

Fig. S1. ^{1}H NMR spectrum of ICz (DMSO-d_6).

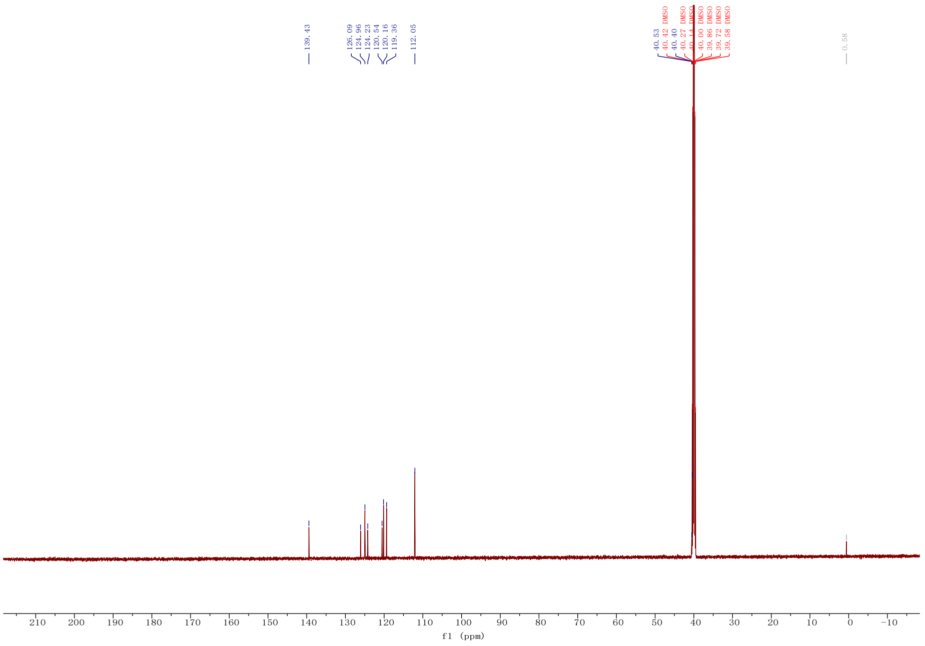

Fig. S2. ^{13}C NMR spectrum of ICz (DMSO-d_6).

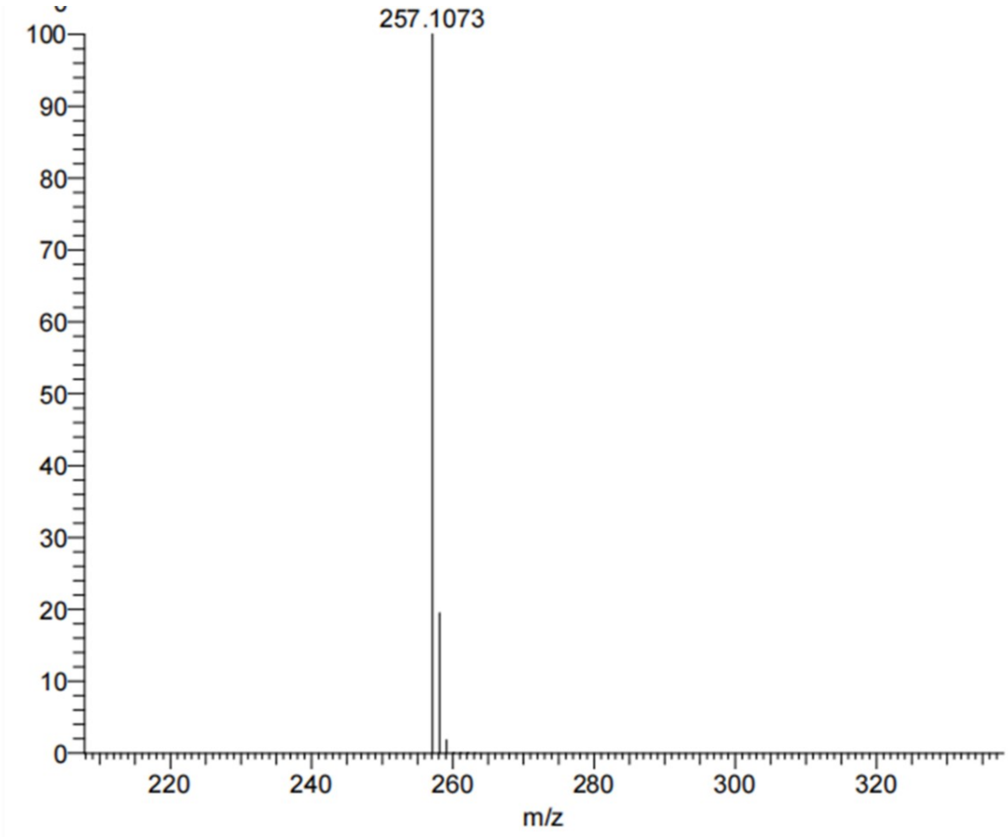

Fig. S3. ESI-MS spectrum of ICz.

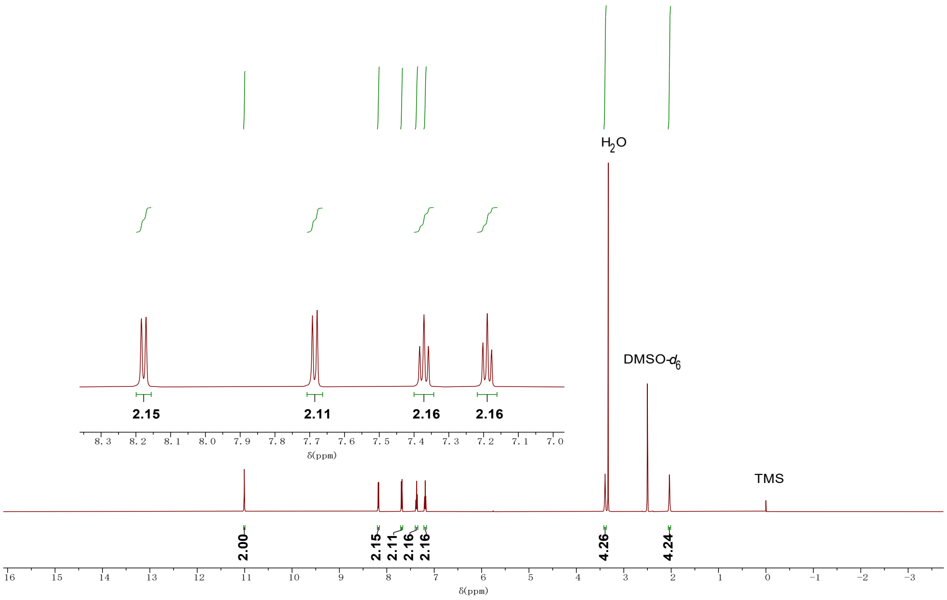

Fig. S4. ^{1}H NMR spectrum of HICz (DMSO-d_6).

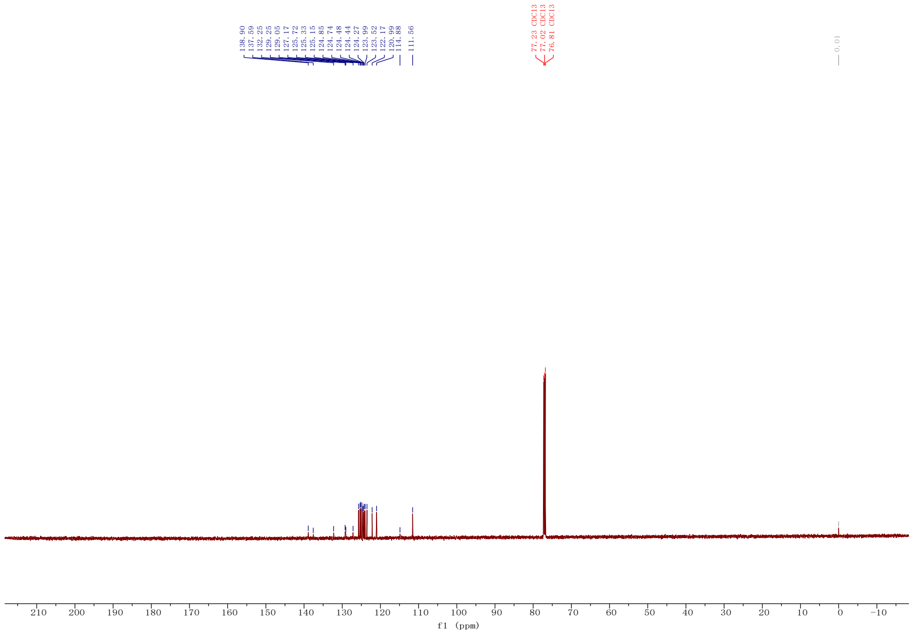

Fig. S5. ^{13}C NMR spectrum of HICz (DMSO-d_6).

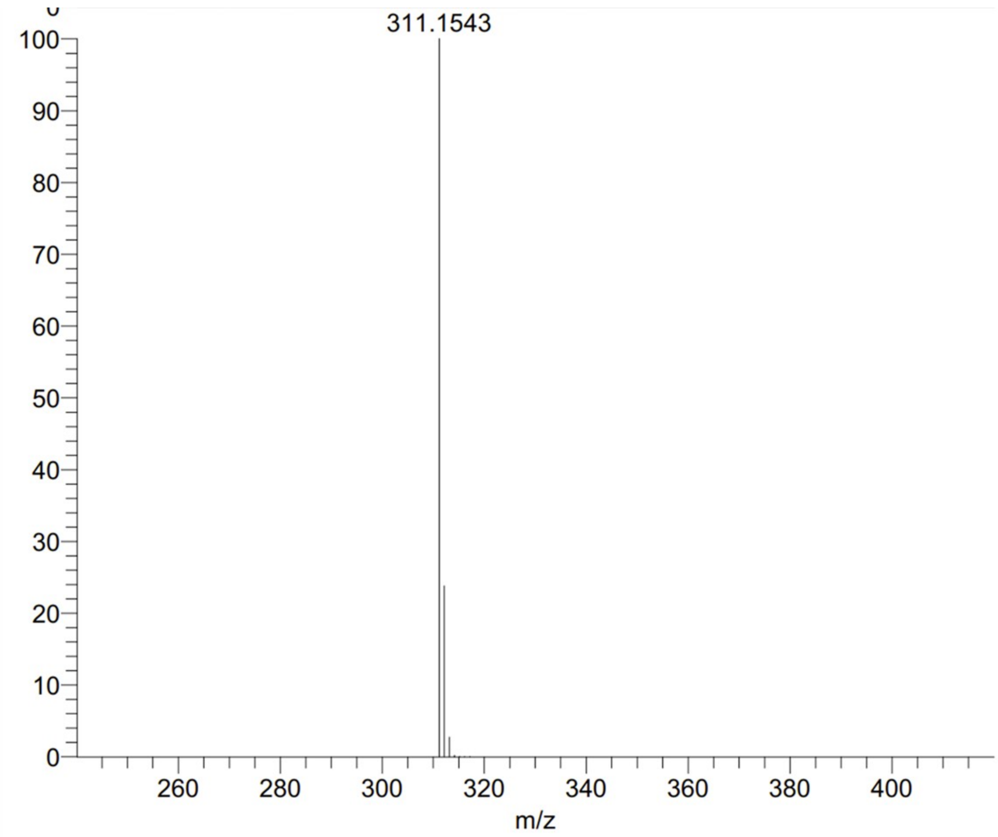

Fig. S6. ESI-MS spectrum of HICz.

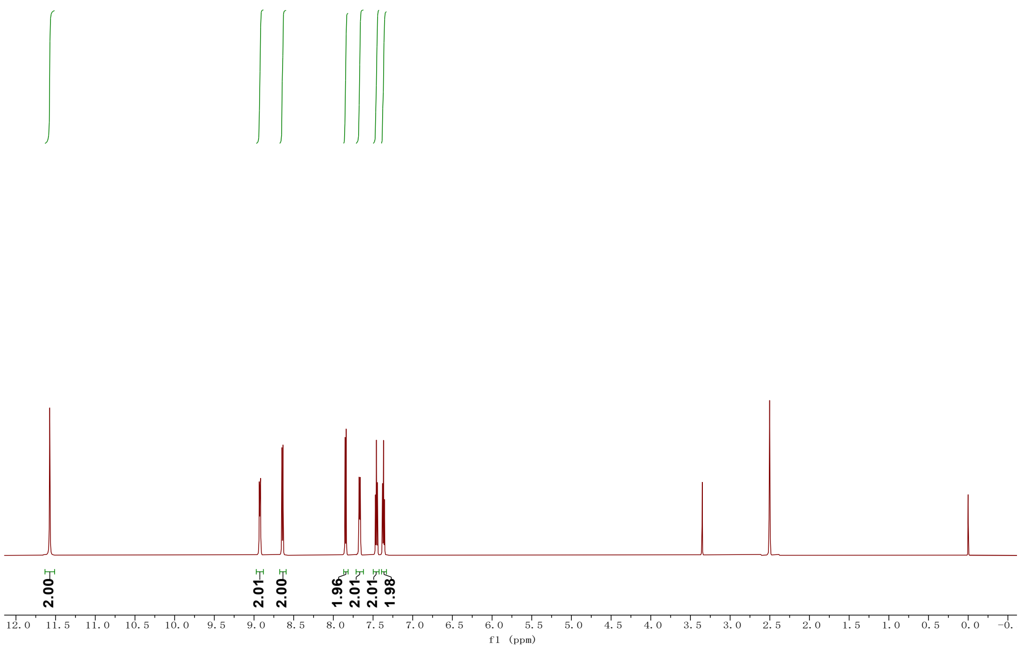

Fig. S7. ^{1}H NMR spectrum of BICz (DMSO-d_6).

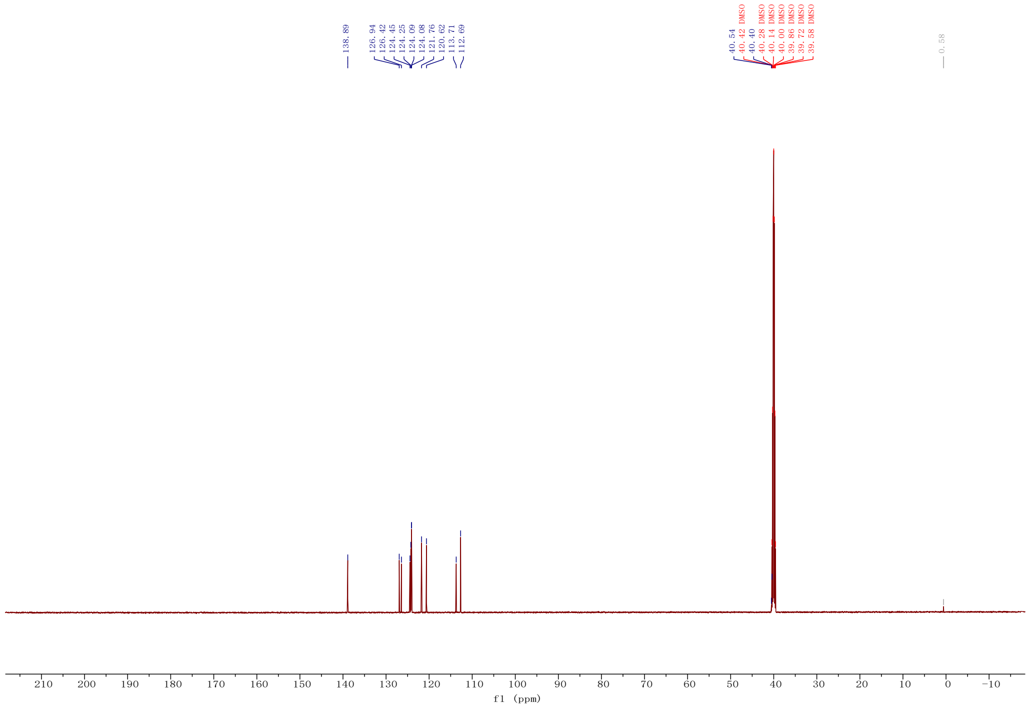

Fig. S8. ^{13}C NMR spectrum of BICz (DMSO-d_6).

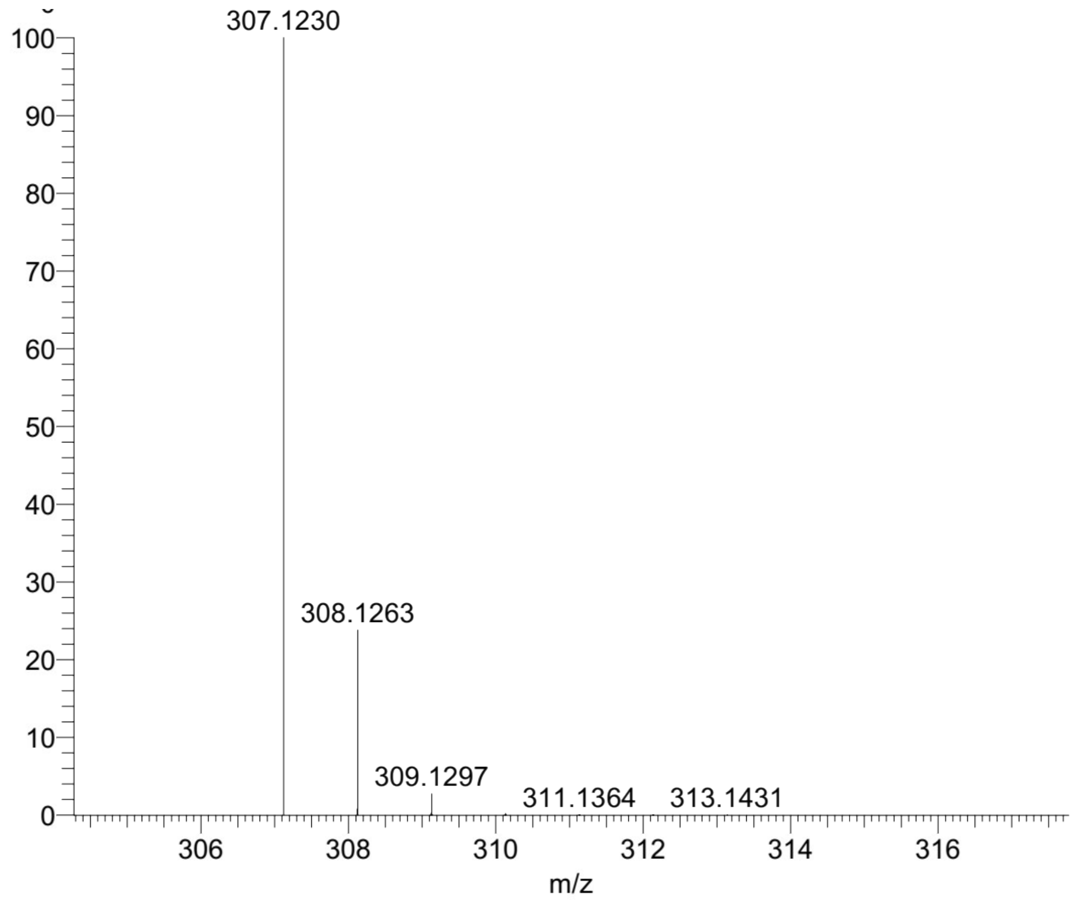

Fig. S9. ESI-MS spectrum of BICz.

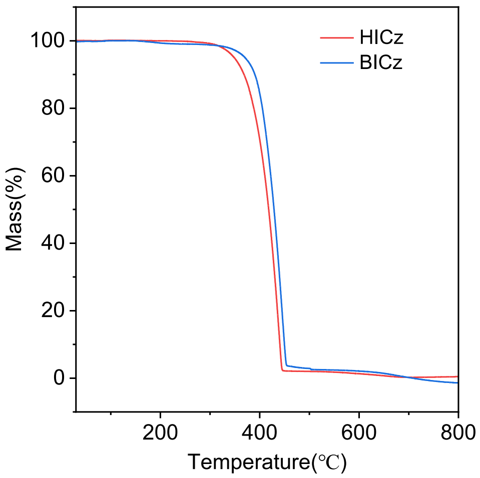

Fig. S10. a) TG curves of HICz and BICz.

4. Photophysical properties of ICz, HICz, and BICz

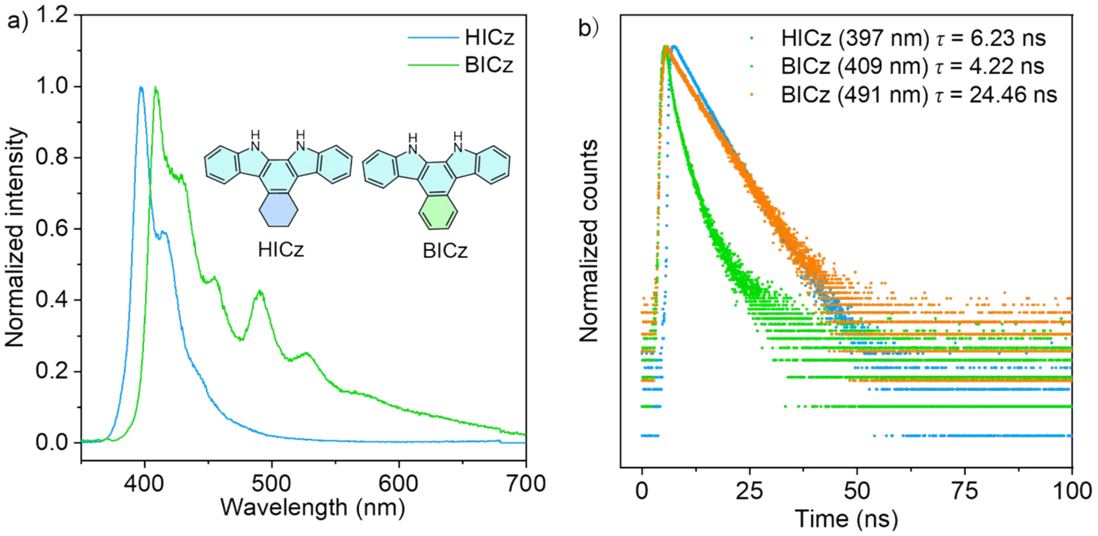

Fig. S11. a) Steady-state PL spectra (λ_{ex}=330 nm) and b) emission decay curves of HICz (λ_{em}=397 nm) and BICz (λ_{em}=409 nm, λ_{em}=491 nm).

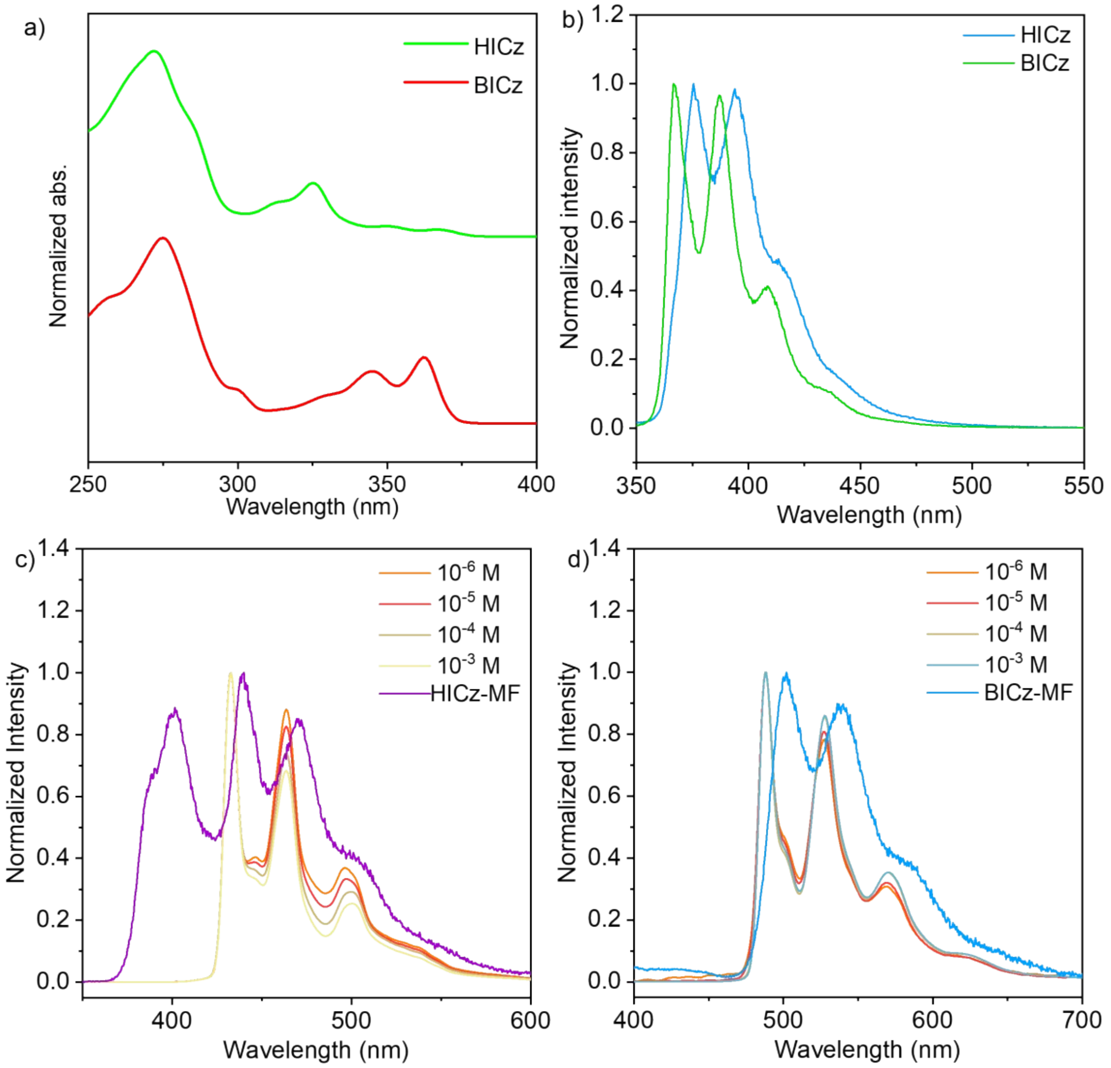

Fig. S12. a) UV-visible absorption spectra of HICz and BICz in THF (Concentration: 10 μM). b) Normalized steady-state PL spectra of HICz and BICz in THF under ambient conditions. c) Delayed PL spectra of HICz in THF solutions at 77 K measured at different concentrations (λ_{ex} = 310 nm) and in MF polymer films under ambient conditions (λ_{ex} = 330 nm). d) Delayed PL spectra of BICz in THF solutions at 77 K measured at different concentrations (λ_{ex} = 310 nm) and in MF polymer films under ambient conditions (λ_{ex} = 365 nm).

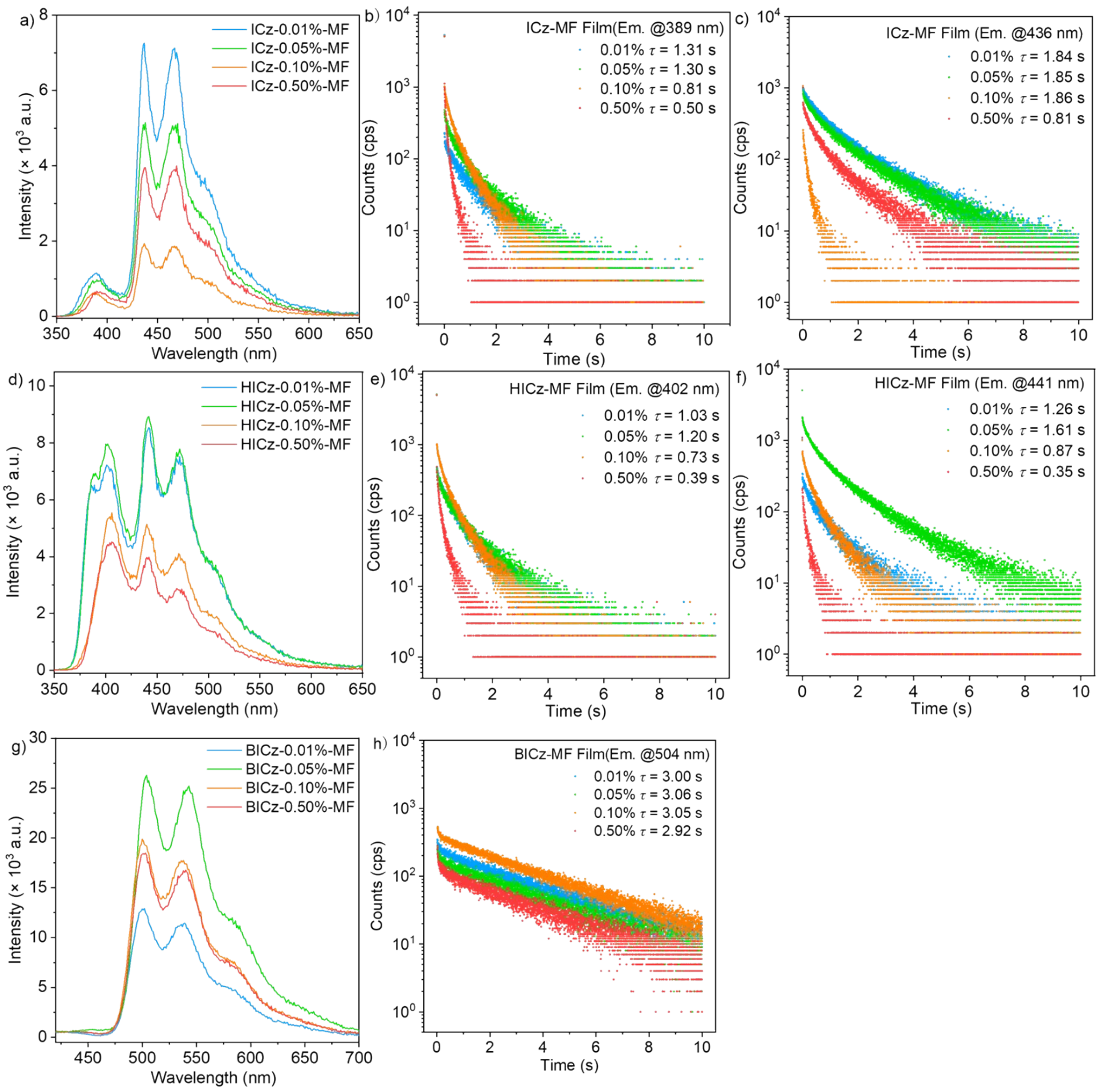

Fig. S13. Delayed spectra of polymer films with different dopant concentrations of a) ICz-MF, d) HICz-MF and g) BICz-MF under ambient conditions. b) Emission decay curves at 389 nm for the ICz-MF films with different dopant concentrations under ambient conditions. c) Emission decay curves at 436 nm for the ICz-MF films with different dopant concentrations under ambient conditions. e) Emission decay curves at 402 nm for the HICz-MF films with different dopant concentrations under ambient conditions. f) Emission decay curves at 441 nm for the HICz-MF films with different dopant concentrations under ambient conditions. h) Emission decay curves at 504 nm for the BICz-MF films with different dopant concentrations under ambient conditions.

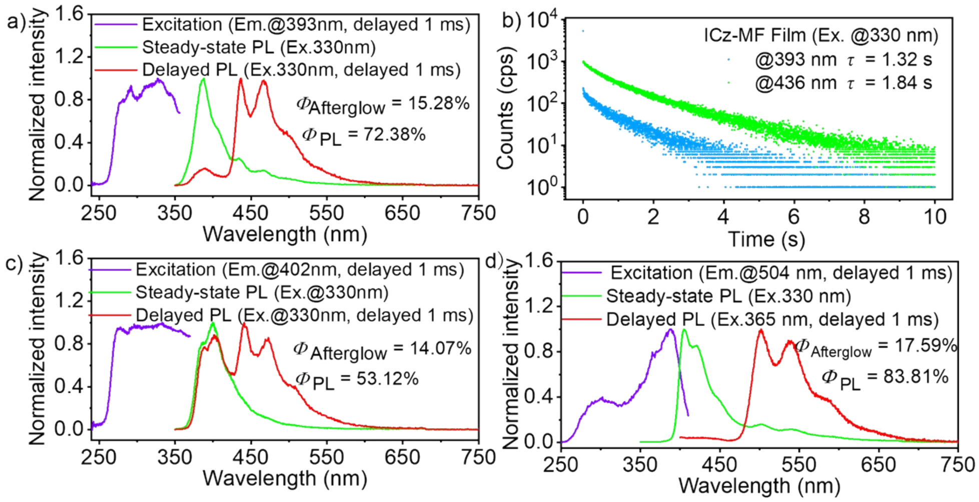

Fig. S14. a) Excitation, steady-state and delayed PL spectra of ICz-MF. b) Delay decay curves of ICz-MF. c) Excitation, steady-state and delayed PL spectra of HICz-MF. d) Delay decay curves of HICz-MF. e) Excitation, steady-state PL spectra, and delayed PL spectra of BICz-MF. f) Delay decay curves of BICz-MF (delayed 1ms, λ_{ex} = 330 nm).

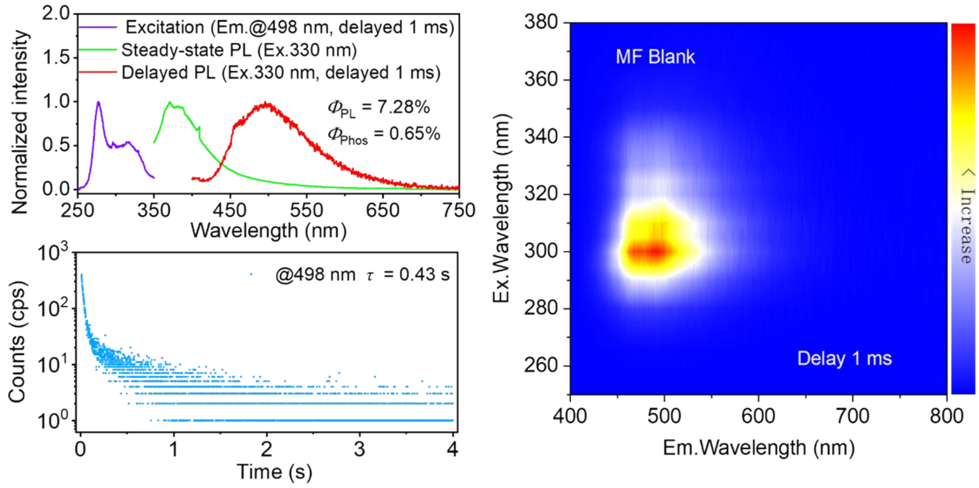

Fig. S15. Photophysical properties of blank MF film under ambient conditions.

Table S4. Photophysical parameters of the doped films.

Doping MF films	Prompt fluorescence		TADF		Phosphorescence		Φ_{PLQY}	$\Phi_{Afterglow}$	Φ_{TADF}	Φ_{Phos}
	λ_{PF} (nm)	τ_{PF} (ns)	λ_{DF} (nm)	τ_{DF} (s)	λ_{P} (nm)	τ_{P} (s)				
ICz-MF	388	8.46	393	1.32	436	1.84	71.38%	15.28%	1.29%	13.99%
HICz-MF	399	9.26	402	1.20	441	1.61	53.12%	14.07%	7.27%	6.80%
BICz-MF	399	7.64	/	/	502	3.05	83.81%	17.59%	/	17.59%

λ_{PF}, prompt fuorescence maximum;

τ_{PF}, prompt fuorescence lifetime;

λ_{DF}, delayed fuorescence maximum;

τ_{DF}, delayed fuorescence lifetime;

λ_{P}, phosphorescence maximum;

τ_{P}, phosphorescence lifetime;

Φ_{PLQY}, total photoluminescence quantum yied;

$\Phi_{Afterglow}$, afterglow quantum yied;

Φ_{TADF}, TADF quantum yied;

Φ_{Phos}, phos-horescence quantum yied.

Table S5. Photophysical parameters of the doped films.

Doping MF films	k_{r}^{PF} ($\times 10^{9}$ s^{-1})	k_{nr}^{PF} ($\times 10^{7}$ s^{-1})	k_{ISC} ($\times 10^{7}$ s^{-1})	k_{r}^{Phos} ($\times 10^{-2}$ s^{-1})	k_{nr}^{Phos} ($\times 10^{-1}$ s^{-1})	k_{RISC} ($\times 10^{-3}$ s^{-1})
ICz-MF	6.63	3.38	1.65	7.60	4.67	7.01
HICz-MF	4.22	5.06	0.73	4.22	5.76	45.16
BICz-MF	8.67	2.12	2.3	5.77	2.70	/

Radiative decay rate of fluorescence $k_r^{PF}= \Phi_{PF} / \tau_{PF.}$

Nonradiative decay rate of fluorescence $k_{nr}^{PF}= (1-\Phi_{PF}-\Phi_{Phos})/ \tau_{PF.}$

Intersystem crossing rate $k_{ISC}=\Phi_{Phos}/\tau_{PF}$

Radiative decay rate of phosphorescence $k_r^{Phos}= \Phi_{Phos} / \tau_{Phos.}$

Nonradiative decay rate of phosphorescence $k_{nr}^{Phos}= (1-\Phi_{Phos}) / \tau_{Phos.}$

Reverse intersystem crossing rate $k_{RISC}=\Phi_{TADF}/\tau_{Phos.}$

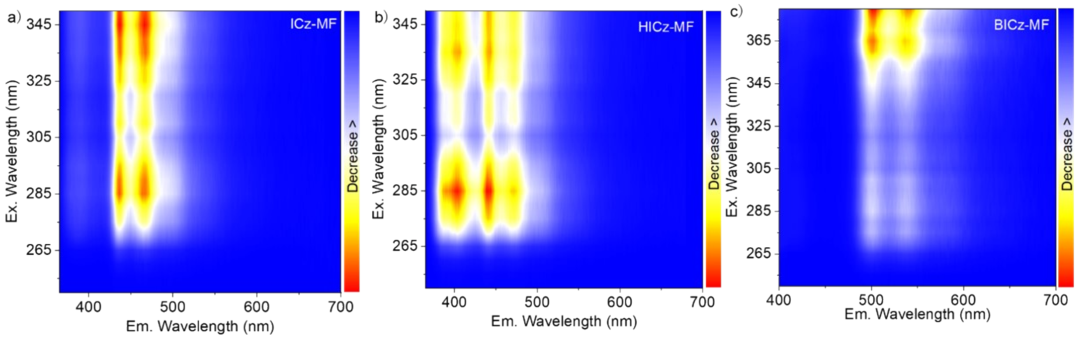

Fig. S16. Excitation-phosphorescence diagram of a) ICz-MF, b) HICz-MF and c) BICz-MF (delayed 1ms).

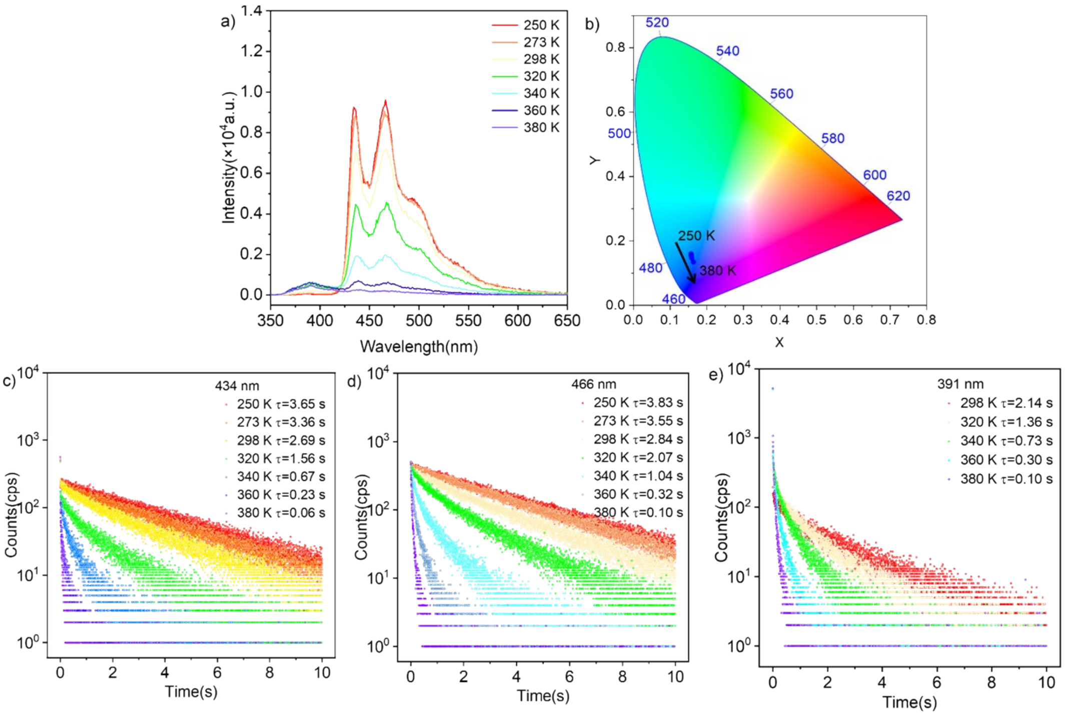

Fig. S17. a) Delayed emission spectrum of ICz-MF at different temperatures under vacuum conditions (Ex: 330 nm) and b) their $CIE_{x,y}$. c) Emission decay curves at 434 nm for ICz-MF at different temperatures in vacuum. d) Emission decay curves at 466 nm for ICz-MF at different temperatures in vacuum. e) Emission decay curves at 391 nm for ICz-MF at different temperatures in vacuum.

Table S6. Photophysical parameters of the doped films.

Excited State	Energy Level (eV)	Transition Configuration	SOC Constant (cm^{-1})	
ICz				
S_1	4.256	H -> L+2 67.8%, H-2 -> L 10.0%	S_0	0.48
T_1	3.540	H-1 -> L 79.4%, H -> L+2 5.5%	S_0	0.09
			S_1	0.13
T_2	3.550		S_1	0.02
T_3	4.131	H -> L+1 60.4%, H-2 -> L 24.8%, H-4 -> L+2 5.1%	S_1	0.17
T_4	4.262	H-1 -> L+1 43.7%, H-3 -> L 14.3%, H -> L+3 8.9%, H-4 -> L 8.7%, H-2 -> L+2 7.6%	S_1	0.05
T_5	4.286	H -> L+2 32.2%, H-2 -> L 26.7%, H-1 -> L 15.1%, H -> L+1 9.9%, H-4 -> L+1 5.5%	S_1	0.15
T_6	4.570		S_1	0.06
T_7	4.756		S_1	0.34
T_8	4.777		S_1	0.00
T_9	4.949		S_1	0.12
T_{10}	5.134		S_1	0.40
HICz				
S_1	4.155	H -> L+1 62.1%, H -> L+2 13.6%, H-1 -> L 10.1%	S_0	0.48
T_1	3.450	H-1 -> L 79.8%, H -> L+2 5.9%	S_0	0.30
			S_1	0.03
T_2	3.504		S_1	0.14
T_3	4.135	H -> L+1 62.9%, H-2 -> L 19.7%	S_1	0.18
T_4	4.233	H-1 -> L+1 36.1%, H-4 -> L 22.4%, H -> L+3 12.3%, H-2 -> L+2 10.7%	S_1	0.12
T_5	4.302	H -> L+2 39.2%, H-2 -> L 27.3%, H-1 -> L 14.7%	S_1	0.06
T_6	4.519		S_1	0.06
T_7	4.711		S_1	0.44
T_8	4.751		S_1	0.04
T_9	4.908		S_1	0.16
T_{10}	5.057		S_1	0.38

Table S7. Photophysical parameters of the doped films.

Excited State	Energy Level (eV)	Transition Configuration	SOC Constant (cm^{-1})	
BICz				
S_1	4.100	H-1 -> L+1 54.9%, H -> L+2 13.3%, H-1 -> L+4 9.0%, H-3 -> L 6.2%	S_0	0.47
T_1	3.069	H -> L 89.9%	S_0	0.43
			S_1	0.01
T_2	3.446		S_1	0.13
T_3	4.059	H -> L+2 41.7%, H-3 -> L 20.2%, H-1 -> L+1 14.3%	S_1	0.07
T_4	4.104	H-2 -> L 76.1%, H-1 -> L+3 7.6%	S_1	0.04
T_5	4.173	H -> L+1 53.2%, H-5 -> L 9.6%, H-4 -> L 7.4%, H-1 -> L+5 5.4%	S_1	0.12
T_6	4.413		S_1	0.00
T_7	4.446		S_1	0.05
T_8	4.480		S_1	0.08
T_9	4.707		S_1	0.06
T_{10}	4.827		S_1	0.06

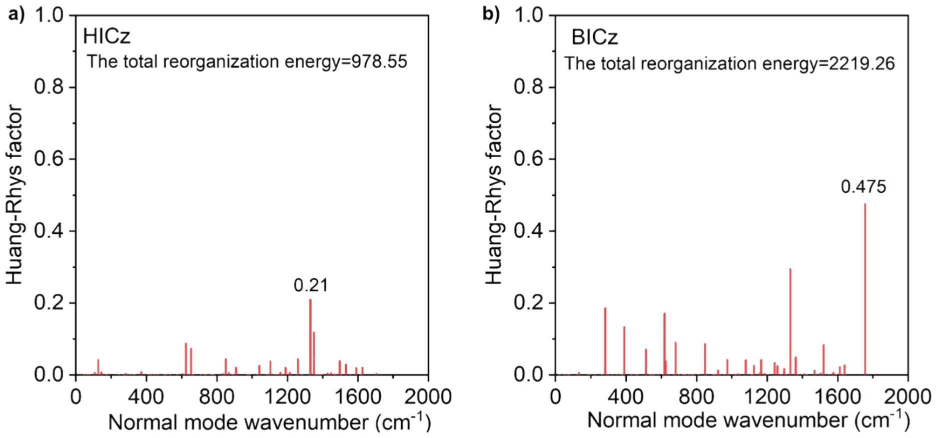

Fig.S18. a) The Huang-Rhys factors versus normal mode wavenumber and the total reorganization energy for T_1-S_0 transition of HICz. b) The Huang-Rhys factors versus normal mode wavenumber and the total reorganization energy for T_1-S_0 transition of BICz.

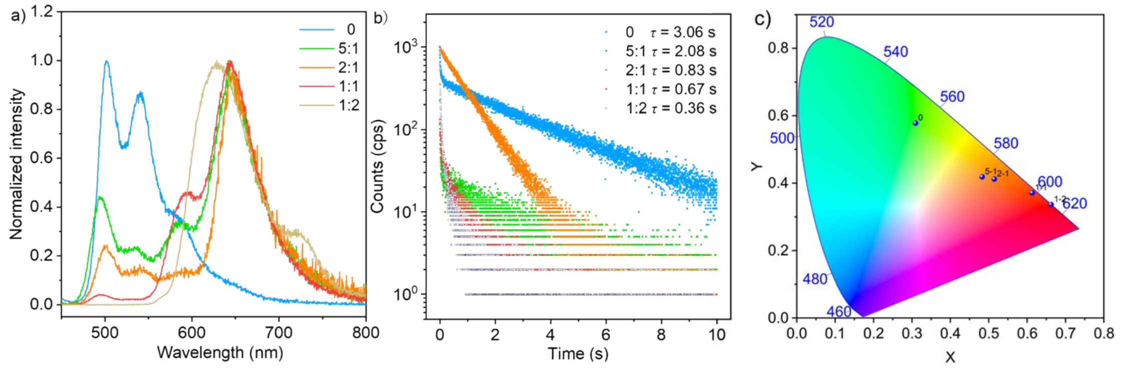

Fig.S19. a) Delayed spectra of BICz-MF@RhB with different mass ratio BICz-MF and RhB under environmental conditions. b) The emission decay curves of BICz-MF@RhB under environmental conditions and c) the corresponding CIE x,y diagram.

Table S8. Photophysical parameters of the BICz-MF@RhB films.

Doping concentration (BICz/RhB)	Phosphorescence		Φ_{PLQY}
	λ (nm)	τ (s)	
5:1	646	2.08	9.27%
2:1	646	0.83	11.78%
1:1	646	0.67	12.28%
1:2	646	0.36	6.12%
1:5	646	0.12	3.44%

5. Supporting Information

1. X. Yao, H. Ma, X. Wang, H. Wang, Q. Wang, X. Zou, Z. Song, W. Jia, Y. Li, Y. Mao, M. Singh, W. Ye, J. Liang, Y. Zhang, Z. Liu, Y. He, J. Li, Z. Zhou, Z. Zhao, Y. Zhang, G. Niu, C. Yin, S. Zhang, H. Shi, W. Huang and Z. An, Ultralong Organic Phosphorescence from Isolated Molecules with Repulsive Interactions for Multifunctional Applications, *Nat. Commun.*, 2022, **13**, 4890.
2. D. Wang, J. Gong, Y. Xiong, H. Wu, Z. Zhao, D. Wang and B. Z. Tang, Achieving Color-Tunable and Time-Dependent Organic Long Persistent Luminescence via Phosphorescence Energy Transfer for Advanced Anti-Counterfeiting, *Adv. Funct. Mater.*, 2023, **33**, 2208895.
3. J. Yu, Z. Sun, H. Ma, C. Wang, W. Huang, Z. He, W. Wu, H. Hu, W. Zhao and W. Zhu, Efficient Visible-Light-Activated Ultra-Long Room-Temperature Phosphorescence Triggered by Multi-Esterification, *Angew. Chem. Int. Ed.*, 2023, **62**, e202316647.
4. J. Zhen, J. Long, X. Guo, Q. Wang and X. Zeng, Tryptophan-Doped Poly(vinyl alcohol) Films with Ultralong-Lifetime Room-Temperature Phosphorescence and Color-Tunable Afterglow Under Ambient Conditions, *Chem-Eur J*, 2024, **30**, e202304137.
5. R. Liu, C. Liu, C. Fu, Z. Zhu, K. Chen, C. Li, L. Wang, Y. Huang and Z. Lu, Ambient Phosphor with High Efficiency and Long Lifetime in Poly(Methyl Methacrylate) Through Charge-Transfer-Mediated Triplet Exciton Formation for Photolithography Applications, *Angew. Chem. Int. Ed.*, 2024, **63**, e202312534.
6. Z. Guan, Z. Tang, Z. Yao, Q. Guo, S. Zhang, Z. Lv, X. Zhang, N. Ma, X. Liu and Z. Hu, "Rigid-Flexible" Strategy Realizes Robust Ultralong Phosphorescence for Multifunctional Display Unit and Photoreceptor Synapse, *Adv. Mater.*, 2025, **37**, e07192.
7. Y. Liang, P. Hu, H. Zhang, Q. Yang, H. Wei, R. Chen, J. Yu, C. Liu, Y. Wang, S. Luo, G. Shi, Z. Chi and B. Xu, Enabling Highly Robust Full-Color Ultralong Room-Temperature Phosphorescence and Stable White Organic Afterglow from Polycyclic Aromatic Hydrocarbons, *Angew. Chem. Int. Ed.*, 2024, **63**, e202318516.
8. H. Zhang, S. Wu, Y. Liang, Z. Zhang, H. Wei, Q. Yang, P. Hu, C. Liu, Z. Yang, C. Zheng, G. Shi, Z. Chi and B. Xu, Enabling Efficient and Ultralong Room-Temperature Phosphorescence from Organic Luminogens by Locking The Molecular Conformation in Polymer Matrix, *Chem. Eng. J.*, 2024, **497**, 154949.
9. L. Xu, H. Wei, G. Xie, B. Xu and J. Zhao, Ultralong MRTADF and Room-Temperature Phosphorescence Enabled Color-Tunable and High-Temperature Dual-Mode Organic Afterglow from Indolo[3,2-b]carbazole, *Adv. Funct. Mater.*, 2024, **34**, 2402428.
10. Y. Yang, Y. Liang, Y. Zheng, J.-A. Li, S. Wu, H. Zhang, T. Huang, S. Luo, C. Liu, G. Shi, F. Sun, Z. Chi and B. Xu, Efficient and Color-Tunable Dual-Mode Afterglow from Large-Area and Flexible Polymer-Based Transparent Films for

Anti-Counterfeiting and Information Encryption, *Angew. Chem. Int. Ed.*, 2022, **61**, e202201820.
11. M. Chen, B. Liu, J. Ren, C. Zhang, Z. Ren and Z. Guan, Stretchable, Ultralong Room-Temperature Phosphorescence Poly(urethane-urea) Elastomer Resistant to Humidity and Heat, *Adv. Mater.*, 2025, **37**, 2504825.
